# Population‐Based Multi‐Omics and Cohort Study Identifying Predictive Biomarkers and Therapeutic Targets for Psoriatic Disease

**DOI:** 10.1002/advs.202514130

**Published:** 2025-12-02

**Authors:** Tianxing Wu, Jialiang Luo, Haoyuan Qiu, Weijie Shao, Yueyang Lu, Meixuan Luo, Zhaofeng Lin, Yan Zhang, Libo Zhang, Hong Wang, Jia Zhou, Guangfeng Ruan, Peihua Cao, Daming Zuo

**Affiliations:** ^1^ Clinical Research Centre Zhujiang Hospital Southern Medical University Guangzhou Guangdong 510260 China; ^2^ Institute of Molecular Immunology Guangdong Provincial Key Laboratory of Immune Regulation and Immunotherapy School of Laboratory Medicine and Biotechnology Southern Medical University Guangzhou Guangdong 510515 China; ^3^ Department of Dermatology Guangdong Medical Products Administration Key Laboratory for Research and Evaluation of Drugs for Inflammatory Diseases the Fifth Affiliated Hospital Southern Medical University Guangzhou Guangdong 510900 China; ^4^ Department of Immunology School of Basic Medical Sciences Southern Medical University Guangzhou Guangdong 510515 China; ^5^ The First School of Clinical Medicine Nanfang Hospital Southern Medical University Guangzhou Guangdong 510515 China; ^6^ Department of Biostatistics School of Public Health Southern Medical University Guangzhou Guangdong 510515 China; ^7^ Clinical Research Centre Guangzhou First People's Hospital Guangzhou Medical University Guangzhou Guangdong 510180 China; ^8^ Department of Rheumatology Guangzhou First People's Hospital Guangzhou Medical University Guangzhou Guangdong 510180 China; ^9^ Key Laboratory of Infectious Diseases Research in South China (Southern Medical University) Ministry of Education Guangzhou Guangdong 510515 China

**Keywords:** biomarker, predictive model, psoriatic disease, si‐RNA, therapy

## Abstract

Psoriatic disease (PsD) is a chronic inflammatory disease, with significant challenges in early risk stratification and drug development. Integration of proteomic and genomic data provides an unprecedented opportunity to identify predictive biomarkers and therapeutic targets for PsD. Here, through systemic genetic analyses, expression validation, and prospective cohort study, CDSN and PRSS8 were identified as candidate biomarkers and potential therapeutic targets for PsD. Individuals with higher levels of CDSN and PRSS8 were nearly three times more likely to develop PsD compared to the general population. It develops prediction models in adults without PsD at baseline from the UK Biobank. Combining CDSN and PRSS8 with demographics produced desirable predictions for PsD (area under the curve (AUC) = 0.80) and exhibited high specificity. Moreover, PRSS8 and CDSN were both predominantly localized in keratinocytes, and in vivo gene silencing of these proteins significantly reduced PsD‐like skin lesions and systemic inflammatory markers. The findings strongly suggested that CDSN and PRSS8 are promising biomarkers for PsD onset and progression, providing a 12‐year risk assessment window and potential as novel therapeutic targets. These results had important implications for screening high‐risk populations and facilitating early intervention for PsD.

## Graphical abstract

Psoriatic disease (PsD) is a chronic skin disease, with challenges in early risk stratification and drug development. Through gene‐level causal inference framework and expression level validation, combined with longitudinal cohort study, CDSN and PRSS8 have been identified as candidate biomarkers and therapeutic targets for PsD. Composite Model based on clinical and protein predictive factors provides a 12‐year risk assessment window. In vivo gene silencing of these proteins significantly reduced PsD‐like skin lesions and systemic inflammatory markers.

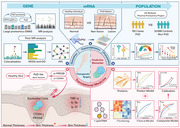

## 1 Introduction

Psoriatic disease (PsD) is a chronic inflammatory disease affecting more than 125 million people worldwide.^[^ ^1^, ^2^ ^]^ Regretfully, there is no cure for PsD at present.^[^ ^3^ ^]^ Existing therapeutic strategies for PsD mainly provide temporary symptomatic relief and are frequently followed by disease relapse.^[^ ^4^ ^]^ Consequently, PsD management guidelines emphasize the critical need for early identification and rigorous validation of biomarkers to aid in predicting treatment responses, monitoring disease activity, and guiding therapeutic decisions.^[^ ^5^ ^]^ However, most biomarkers identified thus far merely indicate a correlation with PsD and tend to act as passive indicators rather than active drivers of disease progression.^[^ ^6^, ^7^ ^]^ Hence, there is an urgent need to discover new biomarkers that not only enable accurate prediction but also serve as therapeutic targets to halt PsD progression.

The human plasma consists of circulating proteins that are secreted by normal cells and those that leak from damaged tissues under pathological conditions.^[^ ^8^ ^]^ These proteins play crucial roles as mediators, indicators, and messengers in inflammatory diseases, serving as essential sources of biomarkers and offering valuable potential as therapeutic targets.^[^ ^9^ ^]^ Notably, previous study has demonstrated significant associations between circulating protein levels and PsD.^[^ ^10^ ^]^ Recent advancements in proteomics have made it possible to simultaneously measure thousands of circulating proteins in large cohorts.^[^ ^11^ ^]^ Furthermore, the integration of genomic and proteomic data through genome‐wide association studies (GWAS) has led to the identification of numerous protein quantitative trait loci (pQTLs).^[^ ^12^ ^]^ By combining pQTLs with disease‐variant associations, researchers can investigate the effects of proteins on disease progression based on gene‐level causal inference framework.^[^ ^13^ ^]^ This approach utilizes pQTLs as instrumental variables for their corresponding proteins, allowing for high‐throughput screening of circulating proteins to establish causal inference with PsD.

In recent years, risk prediction models have been derived, validated, and implemented in clinical practice to predict the incidence of various chronic inflammatory diseases.^[^ ^14^, ^15^ ^]^ However, the application of risk prediction models in PsD remains largely unexplored. As PsD garners increasing attention, the development of risk prediction models could significantly enhance the early classification, diagnosis, and prognosis of the disease.^[^ ^1^ ^]^ Herein, we aimed to address this gap by identifying novel predictive biomarkers and validating their role in disease progression. We employed a comprehensive approach to identify candidate proteins causally associated with PsD. Integrating large‐scale pQTL datasets enables us to make causal inferences about protein‐disease associations genetically. Subsequent validation at the expression level and population cohorts further enhances our confidence in the potential of candidate proteins as biomarkers. To validate the predictive ability of these candidate biomarkers, we leveraged data from the UK Biobank (UKB) to establish a PsD risk prediction model incorporating both clinical and protein predictors.^[^ ^16^ ^]^ Finally, we analyzed clinical PsD samples and conducted in vivo experiments to further investigate the therapeutic efficacy of these promising proteins. Our research not only enhances the understanding of PsD but also lays the groundwork for more targeted approaches to its management and treatment.

## 2 Experimental Section

**Figure 1:**
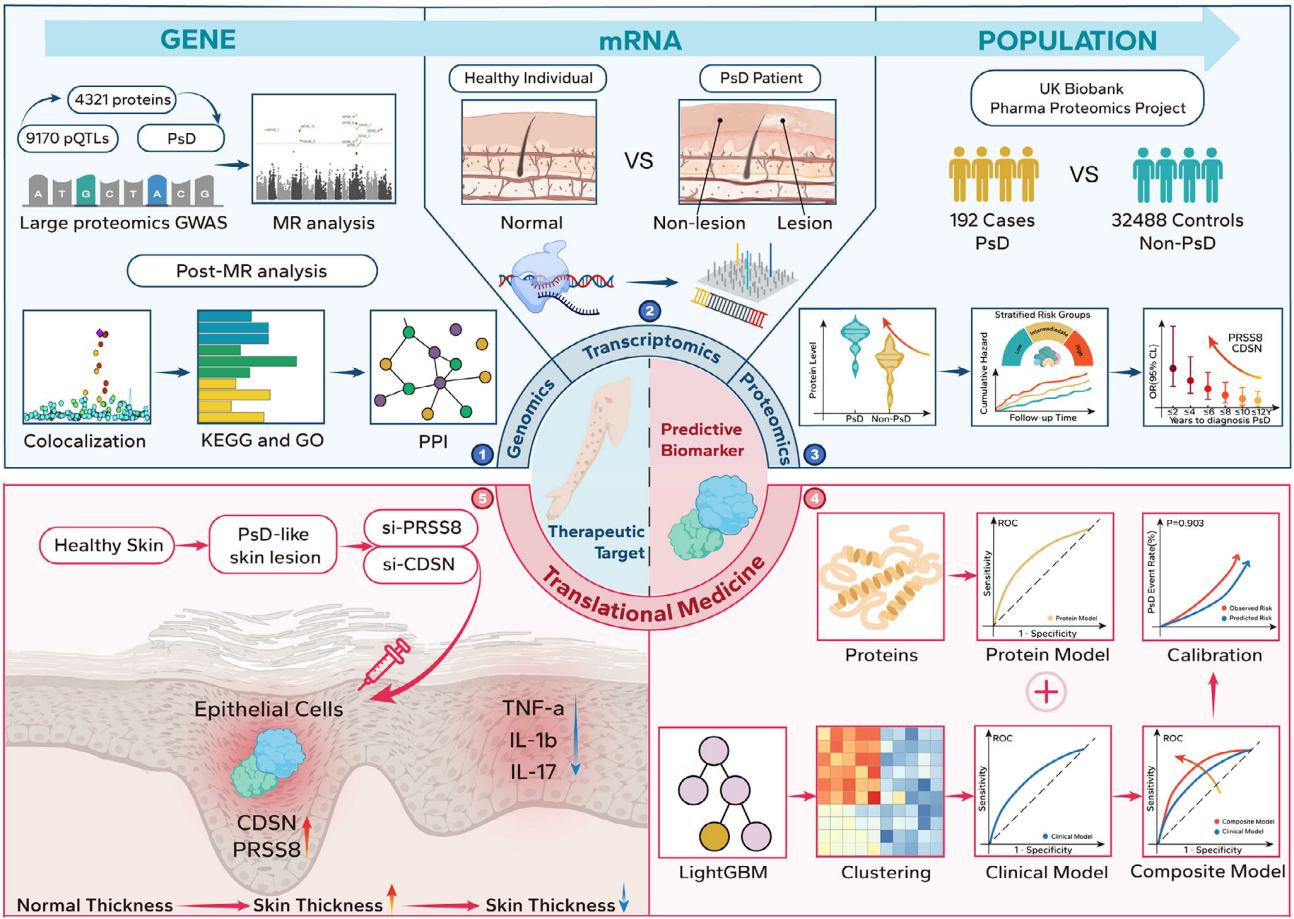
Study Design. The upper part of the figure shows the workflow for screening therapeutic targets and predictive biomarkers. The gene‐level causal inference framework was employed to assess the relationship between plasma proteins and psoriatic disease (Step 1). Gene expression validation and differential analysis were performed at the transcriptional level (Step 2). Subsequently, the prospective associations and predictive capabilities of the candidate proteins with PsD were validated in a longitudinal cohort (Step 3). The lower part focuses on clinical translation, demonstrating that protein biomarkers significantly enhance the predictive performance of the Clinical Model (Step 4). We also validated the therapeutic effects of *CDSN* and *PRSS8* in vivo and in vitro using the siRNA (Step 5). pQTL, protein quantitative trait loci; PsD, psoriatic disease; GWAS, genome‐wide association study; MR, Mendelian randomization; KEGG, Kyoto encyclopedia of genes and genomes; GO, gene ontology; PPI, protein‐protein interaction; CDSN, corneodesmosin; PRSS8, prostasin; LightGBM, light gradient boosting machine; TNF‐α, tumor necrosis factor alpha; IL‐1β, interleukin 1 beta; IL‐17, interleukin 17.

This study aims to identify potential therapeutic targets for PsD, assess their capabilities as predictive biomarkers, and experimentally validate their roles in therapy (**Figure** **1**). To achieve this, we undertook the following five steps: 1) We conducted an  integration of genome‐wide associations and constructed the largest known plasma protein pQTL database. Subsequently, genetically predicted proteins associated with PsD were identified. Further sensitivity analyses, colocalization analyses, and functional analyses deepened our understanding of the relationships between the candidate proteins and PsD. 2) We compared the mRNA expression levels of these candidate proteins in normal donors, lesional and non‐lesional skin from PsD patients. 3) The predictive capability of these candidate proteins was prospectively assessed more than 12 years using data from the UKB. We further validated the specificity of the protein biomarkers and investigated their association with both the activity and severity of PsD. 4) Additionally, we conducted a comprehensive screening of clinical predictors for PsD using the machine learning method and developed the first predictive model that integrates both clinical and protein predictors. 5) To further demonstrate the therapeutic potential of two proteins, CDSN and PRSS8, we silenced their expression using small interfering RNA (siRNA) in an imiquimod‐induced PsD‐like skin lesion model in mice.

**Step 1: Effects of proteins on PsD based on gene‐level causal inference framework**

### 2.1 Proteomic Data Sources

Genetic instruments for circulating proteins were derived from nine independent proteomics genome‐wide association studies.^[^ ^12^, ^16^, ^17^, ^18^, ^19^, ^20^, ^21^, ^22^, ^23^ ^]^ The selection of these studies was based on predefined criteria requiring a minimum sample size exceeding 500 participants and measurement of more than 50 distinct proteins. Detailed characteristics of each proteomics GWAS, including study population demographics, protein measurement platforms, sample sizes, and data collection periods, are presented in Table S1 (Supporting Information). All proteomic studies were conducted in populations of European ancestry to maintain genetic homogeneity with the outcome dataset and minimize potential bias from population stratification.

### 2.2 Outcome Data Source

Summary statistics for PsD were obtained from the largest available European GWAS meta‐analysis to date, comprising 15967 cases and 28194 controls, totaling 44161 individuals.^[^ ^24^ ^]^ The diagnostic criteria for PsD cases followed established clinical guidelines, encompassing both psoriasis and psoriatic arthritis phenotypes. Complete details regarding the outcome data source, including study design, participant recruitment periods, case ascertainment methods, and control selection criteria, are provided in Table S1 (Supporting Information).

### 2.3 Selection and Quality Control of Genetic Variants

The selection of protein quantitative trait loci (pQTL) as genetic instruments followed a rigorous multi‐step protocol. First, single nucleotide polymorphisms associated with protein levels were identified based on *P*‐value thresholds recommended in the respective source studies, as detailed in Table S1 (Supporting Information). Second, variants located within the major histocompatibility complex region on chromosome 6 (26–34 Mb) were systematically excluded due to the complex linkage disequilibrium patterns and high gene density in this region, which could introduce horizontal pleiotropy. Third, linkage disequilibrium clumping was performed using thresholds of r^2^ greater than 0.01 within a 5 000 kb window to identify independent pQTLs for each protein. Fourth, genetic instruments associated with five or more proteins were excluded to minimize potential pleiotropic effects.

The analysis focused specifically on cis‐acting pQTLs, defined as genetic variants located within a 1,000 kb window of the corresponding protein‐coding gene. This approach was adopted to minimize the influence of horizontal pleiotropy, as cis‐pQTLs are more likely to exert their effects through direct regulation of the nearby gene rather than through alternative biological pathways. The characteristics of all selected cis‐pQTLs are documented in Table S3 (Supporting Information).

For variants present in the proteomic GWAS but absent from the PsD GWAS summary statistics, missing information was imputed using the 1000 Genomes European reference panel to ensure comprehensive coverage. All genomic analyses utilized the GRCh38/hg38 reference genome build, representing the current standard and providing the most comprehensive genomic annotation.

### 2.4 Ethics and Informed Consent

This study exclusively utilized summary‐level data from published genome‐wide association studies. All original studies received approval from their respective institutional ethics committees, and participants provided informed consent for genetic research. As no individual‐level data were accessed in the present analysis, separate ethical approval was not required.

### 2.5 Assumptions

The validity of Mendelian randomization rests upon three core instrumental variable assumptions. First, the relevance assumption requires that the genetic variants are robustly associated with the exposure (circulating protein levels). Second, the independence assumption stipulates that the genetic variants are independent of confounders affecting both the exposure and outcome. Third, the exclusion restriction assumption mandates that the genetic variants influence the outcome (PsD) exclusively through their effects on the exposure, with no alternative pathways. In the context of this study, these assumptions translate to requiring that the selected pQTLs demonstrate strong associations with their corresponding proteins, are not associated with confounders of the protein‐PsD relationship, and affect PsD risk only through alterations in protein levels rather than through pleiotropic mechanisms.

Additional assumptions underlying the sensitivity analyses include the InSIDE (Instrument Strength Independent of Direct Effect) assumption for MR‐Egger regression, which requires that the strength of the genetic instruments is independent of their direct effects on the outcome. For colocalization analyses, the assumption is that a shared causal variant underlies both the protein association and the PsD association within the genomic region examined.

### 2.6 Statistical Methods for the Main Analysis

The primary analytical approach employed two‐sample Mendelian randomization to estimate the causal effect of genetically predicted protein levels on PsD risk. Genetically predicted protein levels served as the exposure variable, while PsD case‐control status represented the outcome. For proteins instrumented by a single pQTL, causal estimates were derived using the Wald ratio method, calculated as the ratio of the SNP‐outcome association to the SNP‐exposure association. When two or more independent pQTLs were available for a given protein, the inverse variance weighted method was applied as the primary estimator, which provides a precision‐weighted average of the individual variant ratio estimates under the assumption of balanced horizontal pleiotropy.

Genetic variants were handled as continuous variables representing allele dosages in the original GWAS. Effect estimates and standard errors for both exposure and outcome associations were extracted from the summary statistics. The weights for combining multiple instruments in the inverse variance weighted method were determined by the inverse of the variance of each SNP‐outcome association, thereby giving greater influence to more precisely estimated effects.

Protein levels were analyzed on their original scales as reported in the source proteomics GWAS, with effect estimates representing the change in log odds of PsD per standard deviation increase in genetically predicted protein level. Missing data for genetic variants were addressed through imputation using the 1000 Genomes European reference panel as the reference dataset, ensuring harmonization between exposure and outcome summary statistics.

To account for multiple testing across the numerous proteins examined, a Bonferroni‐corrected significance threshold was applied. Proteins demonstrating associations surpassing this multiple‐testing threshold were carried forward for subsequent colocalization and functional analyses.

### 2.7 Estimate the Impact of Cohort Overlap

We evaluated the potential bias due to sample overlap in MR. Sample overlap between the exposure and outcome GWAS can bias the causal estimate either towards the null or in the direction of the observational association between the risk factor and outcome. Relative bias, which quantifies the extent to which MR's causal estimate is biased due to sample overlap relative to the observational estimate^1^, is calculated as:

(1) $$\mathrm{Relative}\;\mathrm{bias}=\phi \times \frac{1}{F}$$

where *φ* is the proportion of the sample overlap (ranging from 0 to 1) and *F* is the F‐statistic of the exposure. We estimated the causal effect of protein levels on PsD. The median F‐statistic for protein levels was 254.76 (25th percentile: 102.33, 75th percentile: 659.19, minimum: 26.95). The maximum potential sample overlap between pQTL and outcome GWAS was 4612, reflecting the number of individuals available in the proteomic data. Therefore, the percentage of potential sample overlap for PsD was calculated as 10.44% (4612/44161). Using the minimum F‐statistic and the maximum possible sample overlap, the estimated maximum relative bias was approximately 0.3874%. Overall, this relative bias is considered acceptable given the robust genetic instruments and minimal overlap.

### 2.8 Sensitivity Analyses and Additional Analyses

A comprehensive suite of sensitivity analyses was conducted to evaluate the robustness of the main findings and to address potential violations of the instrumental variable assumptions. The weighted median method was applied as an alternative estimator that provides consistent estimates when at least 50 percent of the weight in the analysis derives from valid instruments, thereby offering protection against some invalid instruments. This method was particularly employed when heterogeneity across multiple instruments was detected using Cochran's Q test.

MR‐Egger regression was implemented to detect and adjust for potential directional horizontal pleiotropy. This method provides a valid estimate of the causal effect under the InSIDE assumption, even in the presence of pleiotropy, and tests for directional pleiotropy through the intercept term. A non‐zero intercept provides evidence of horizontal pleiotropy, while the slope coefficient represents the pleiotropic‐corrected causal estimate.

The MR‐PRESSO (Mendelian Randomization Pleiotropy RESidual Sum and Outlier) framework was utilized to formally detect and correct for outlier variants that may introduce horizontal pleiotropy. This method identifies variants with disproportionate contributions to heterogeneity and provides corrected causal estimates after their removal. The analysis was conducted with 10000 permutations to ensure robust outlier detection.

To investigate potential reverse causation, whereby PsD might influence protein levels rather than the converse, bidirectional Mendelian randomization analyses were performed for all proteins showing significant associations in the forward direction. In these reverse analyses, PsD served as the exposure and each candidate protein as the outcome. Genetic instruments for PsD were selected at genome‐wide significance (P less than 5 × 10^−^⁸) and subjected to linkage disequilibrium pruning using identical criteria as the forward analyses (r^2^ less than 0.001 within a 10,000 kb window). The inverse variance weighted method served as the primary approach, supplemented by weighted median, MR‐Egger, simple mode, and weighted mode methods. Non‐significant reverse associations across all methods would support unidirectional causation from proteins to PsD.

Steiger filtering was additionally applied to assess the directionality of the causal relationship by comparing the variance explained in the exposure versus the outcome by the genetic instruments. Results were categorized as true directionality when the instruments explained more variance in the exposure than in the outcome with *P*‐value less than 0.05, false when the reverse was observed, and uncertain when *P*‐value was 0.05 or greater. Proteins yielding false or uncertain directionality results were excluded from further consideration to ensure causal inference validity.

For proteins surpassing the multiple‐testing threshold, colocalization analyses were conducted to determine whether the protein and PsD associations were driven by the same underlying causal variant rather than distinct variants in linkage disequilibrium. Both Bayesian colocalization and Sum of Single Effects (SuSiE) colocalization methods were implemented.^[^ ^25^, ^26^ ^]^ The posterior probability for hypothesis four (H4), representing a shared causal variant between the protein and PsD associations, was calculated. A threshold of PH4 equal to or exceeding 0.8 in at least one method was considered evidence of colocalization, providing strong support that the protein genuinely influences PsD risk through the identified genetic variant.

To contextualize the biological relevance of the identified proteins, protein‐protein interaction networks were constructed using the STRING database version 11.5, which integrates known and predicted protein associations from various sources including experimental data, computational prediction, and text mining. Additionally, functional enrichment analyses were performed to identify overrepresented Gene Ontology biological processes and Kyoto Encyclopedia of Genes and Genomes pathways among the MR‐prioritized proteins, thereby elucidating the biological mechanisms through which these proteins may contribute to PsD pathogenesis.^[^ ^27^, ^28^ ^]^

### 2.9 Software

All Mendelian randomization analyses were conducted using the TwoSampleMR R package (available at http://github.com/MRCIEU/TwoSampleMR) within the R statistical computing environment. MR‐PRESSO analyses utilized the MRPRESSO package version 1.0. Colocalization analyses employed standard Bayesian colocalization methods and the SuSiE framework. Protein‐protein interaction network construction was performed using STRING database version 11.5. Functional enrichment analyses were conducted using the ClusterProfiler R package. Linkage disequilibrium clumping and reference panel operations utilized PLINK software with the 1000 Genomes European reference panel.

**Step 2: Expression of candidate proteins at the transcriptional level**

We extracted the datasets GSE14905 and GSE30999 from the GEO database to investigate the transcriptional expression differences of candidate proteins. The GSE14905 dataset comprised skin samples from 21 normal donors, lesional skin from 29 PsD patients, and non‐lesional skin from 26 PsD patients.^[^ ^29^ ^]^ Group comparisons were performed using one‐way ANOVA. The GSE30999 dataset included lesional and non‐lesional skin samples from 85 paired PsD patients, with paired t‐tests employed for inter‐group comparisons.^[^ ^30^ ^]^

**Step 3: Further evaluation based on population and protein levels**

### 2.10 Study Design and Population

The UK Biobank (UKB) is a large‐scale, prospective cohort study that includes data from over 500,000 participants aged 40 to 69, who were recruited between 2006 and 2010 across the UK (https://www.ukbiobank.ac.uk/). Health care records, both inpatient and outpatient, along with other health‐related information, were collected and regularly updated for all participants. We conducted a longitudinal cohort study of incident PsD based on the UK Biobank Pharma Proteomics Project (UKB‐PPP) to prospectively evaluate the predictive capability of candidate proteins.^[^ ^16^ ^]^ The UKB‐PPP characterized plasma proteomic profiles at baseline, with the majority of samples randomly selected from the UKB cohort. We excluded participants with missing data on candidate proteins or clinical characteristics (n = 18708), missing follow‐up records (n = 711), a self‐reported history of PsD or missing disease history (n = 693), unknown ethnicity (n = 165), and those using biologics or immunosuppressants such as cyclosporine or methotrexate (n = 1262). Ultimately, we included 32680 participants without PsD at baseline, with a median follow‐up of 12.4 years. All participants provided informed consent, and the UKB's scientific protocol and operational procedures were reviewed and approved by the North West Research Ethics Committee (reference no. 06/MRE08/65). We accessed the UKB data under application number 67654 and 131591.

### 2.11 Protein Assessment

We obtained data on plasma proteins using the Cardiometabolic, Inflammation, Neurology, and Oncology 384‐plex panels on the Olink Explore 3072 platform at Olink's facilities in Uppsala, Sweden. All samples were randomized and plated by the UKB laboratory team prior to delivery. The samples were processed using three NovaSeq 6000 Sequencing Systems and Proximity Extension Assay technology. Extensive quality control and normalization of protein concentrations were conducted at Olink's facilities, resulting in Normalized Protein eXpression (NPX) values for each protein per participant. To ensure comparability, we standardized the NPX values of each protein using rank‐based inverse normal transformation. A detailed description of the quality measures for the UKB proteomics dataset has been submitted.^[^ ^16^ ^]^

### 2.12 Outcomes in Cohort Study

PsD was identified using International Classification of Diseases (ICD) and self‐reported codes, with the specific extraction codes detailed in Table S21 (Supporting Information). To mitigate potential bias with participants having PsD without diagnoses at the time of blood collection, which might affect plasma protein concentrations, the observation period for PsD was adjusted to begin from one year after baseline. To further ensure the specificity of the proteins for PsD prediction, data on other diseases that need to be differentiated from PsD were also extracted as outcomes, with the corresponding codes provided in Figure S4.

### 2.13 Statistical Analysis

Categorical variables were analyzed using the χ^2^ test, while continuous data were analyzed using the t‐test. Two‐tailed *P*‐value < 0.05 was considered statistically significant. PsD cases were stratified by time to diagnosis from baseline into the following intervals: 0–2 years, 2–5 years, 5–10 years, and >10 years. For each time window, the Z‐scores for CDSN and PRSS8 expression levels were calculated. Standardized Z‐scores for plasma expression levels were derived by subtracting the mean expression level of the control population from each individual's measured protein level and dividing by the standard deviation of the control population. This process standardized protein measurements across different assay batches, allowing for direct comparison across time intervals. The resulting dimensionless Z‐scores represent the number of standard deviations each measurement deviated from the control mean. Results were visualized as line plots, with controls represented at time zero. Jonckheere‐Terpstra trend tests were performed to assess differences in plasma protein levels over time for individuals with incident PsD across the specified intervals before diagnosis.

All analyses were conducted using standardized protein levels, while raw, unadjusted protein levels were used for visual representations such as Kaplan‐Meier curves. The expression levels of candidate biomarkers were categorized into tertiles, representing high, medium, and low expression groups. Kaplan‐Meier curves were employed to illustrate the risk stratification capability of the protein biomarkers,^[^ ^30^ ^]^ with group comparisons conducted using the Log‐rank test.^[^ ^31^ ^]^ The associations between candidate proteins per 1‐standard deviation (SD) higher measure and incident PsD diagnosis at 2, 4, 6, 8, 10, and 12 years from baseline were evaluated using multivariable Cox proportional hazards regression, adjusting for age, sex, body mass index (BMI), and batch of protein assay. We further adjusted the model for various confounding factors, including medication use (such as medications for cholesterol, blood pressure, diabetes, or the use of exogenous hormones), self‐rated health (as an indicator of comorbidity burden), polygenic risk scores for PsD, and socioeconomic factors (Townsend Deprivation Index). The discriminatory capacity of candidate proteins for predicting the incidence of PsD was evaluated using the area under the receiver operating characteristic (ROC) curve (AUC). Restricted cubic splines (RCS) analysis was used to evaluate the dose‐response relationships between CDSN, PRSS8 and incident PsD risk with 3 knots at the 10th, 50th, and 90th percentiles. The evaluation of additive interactions between CDSN and PRSS8 for PsD risk was conducted using the R package “interaction”. Additive interactions were assessed through the relative excess risk due to interaction (RERI), attributable proportion (AP), and synergy index (S). To account for death as a competing event during the extended follow‐up period, we performed competing risk analysis using the Fine‐Gray subdistribution hazard model implemented in the cmprsk package (version 2.2‐11) in R. Incident PsD was the event of interest, death without PsD was treated as a competing event, and patients alive without PsD were censored. Subdistribution hazard ratios (sHRs) were estimated after adjusting for age, sex, BMI, and batch effects. The 1:1 PS‐matching method was applied between groups with and without PsD based on confounding factors (age, sex, and BMI), and the nearest‐neighbor method with a caliper was set at 0.01. The associations between biomarker levels and PsD risk were examined in the PS‐matched cohort using the Cox proportional hazards model.

### 2.14 Assessment of CDSN and PRSS8 in Relation to PsD Activity

Given the critical roles of IL‐17 and IL‐22 in driving systemic inflammation, and potentially triggering the PsD progression, we examined their relationships with PRSS8 and CDSN. This case‐control analysis utilized Pearson correlation to explore these relationships in PsD patients with joint damage based on data from the UKB‐PPP. Furthermore, we depicted and compared the dynamic expression levels of CDSN and PRSS8 in PsD patients before and after treatment with the biologics etanercept and brodalumab, using data from the GEO database (GSE11903 and GSE53552). GSE11903 includes 15 moderate‐to‐severe PsD patients who were administered etanercept (Amgen) every two weeks for 12 weeks (baseline, and weeks 1, 2, 4, and 12). GSE53552 includes 25 patients with moderate‐to‐severe PsD patients who received a single dose of brodalumab treatment. Biopsies were obtained from a single non‐lesional site at baseline, as well as from three locations within a single predesignated lesion at baseline, day 8, day 15, and day 43.

### 2.15 Assessment of CDSN and PRSS8 in Relation to PsD Severity

PsD patients samples in this section were approved by the Committees for the Ethical Review of Research Involving Human Subjects at the Guangzhou Institute of Dermatology. The characteristics of PsD patients are presented in Table S20 (Supporting Information). PASI score (Psoriasis Area and Severity Index) is a standardized tool used to assess the severity of skin damage in PsD patients. It calculates a total score by evaluating the area and severity of lesions (redness, sweling, and scaling), with higher scores indicating greater severity of the disease. After rehydration of PsD patients sections, antigen retrieval was performed on the sections by heating them in a citrate buffer (pH 6.0) at 100 °C for 10 min. The subsequent procedures were conducted as previously described.^[^ ^32^ ^]^ Paired t‐tests were employed for inter‐group comparisons, and linear correlation analysis was also performed between CDSN, PRSS8, and PASI scores.

**Step 4: Development and evaluation of the PsD prediction model**

### 2.16 Candidate Clinical Features

For the UKB, the UKB‐PPP sub‐cohort was randomly selected from the total cohort and consists of approximately 50000 participants. Therefore, the non‐UKB‐PPP cohort and the UKB‐PPP cohort can be used as the training and validation cohort, respectively. For the clinical prediction model, we used the training cohort to develop the predictive model and to determine the corresponding tuning parameters. The validation cohort was used exclusively to evaluate the model's performance.

We applied a relatively loose inclusion criterion to avoid overlooking any potential associations, initially considering all clinical features collected during baseline visits. Non‐informative variables with more than 40% missing data across participants were excluded. Additionally, procedure‐related variables, such as biological sample processing metrics, diagnosis codes, and device IDs deemed clinically irrelevant, were manually excluded. Ultimately, 289 features were analyzed, encompassing demographic characteristics (n = 3), physical measures (n = 55), biological sample assays (n = 62), and touchscreen‐recorded lifestyle and health information (n = 169). Missing values were not imputed during the screening phase, as the light gradient boosting machine (LightGBM) algorithm inherently supports missing data. This tree‐based model automatically learns split directions for missing values during training.

### 2.17 Clinical Predictors Identification

LightGBM algorithm (https://github.com/microsoft/LightGBM) is an ensemble learning method that typically combines multiple base learners, most commonly decision tree models. LightGBM begins with a weak base learner (a decision tree) and sequentially trains each new tree to correct the errors of the previous ones. Predictor ranking, also known as feature importance ranking, reflects each predictor's ability to identify the future incidence of PsD.

In the first step, we trained 1000 models across various parameter spaces and selected the top 5% (50 out of 1000) based on their area under the ROC curves (AUCs). The clinical factors we selected to construct the predictive model are detailed in Table S22 (Supporting Information), and the hyperparameter search space is detailed in Table S23 (Supporting Information). To ensure equitable contribution from the 50 candidate models, their importance scores were normalized to a range of [0, 1]. The final importance ranking was calculated by averaging the scores of the highest‐performing models and sorting them accordingly. In the second step, the top 50 features were selected based on their ranking. Hierarchical clustering was conducted using Spearman rank‐order correlations to mitigate multicollinearity. An threshold of 1.00 was then applied to prune the dendrogram, retaining only one predictor from each cluster where predictors fell below this threshold. In the third step, a sequential forward selection strategy was employed. Features within the pre‐selected subset were re‐ranked using a newly developed classifier, and consecutive classifiers were built by sequentially adding predictors based on the updated importance rankings. Ultimately, ten predictors were identified for model development. In the fourth step, we used SHapley Additive exPlanations (SHAP) plot to visualize the contribution of each predictor to incident PsD. Given the limited interpretability of machine learning algorithms, we further assessed the epidemiological significance of these clinical predictors by including the ten selected predictors in a multivariable Cox proportional hazards regression model and examining their associations with incident PsD. For continuous variables, HRs and the corresponding 95% confidence intervals (CIs) per 1‐SD higher measure were calculated. The reference for the predictive factor of being born in England is being born outside of the UK.

### 2.18 Predictive Model Development

We implemented LightGBM to develop a clinical risk prediction model incorporating the ten identified predictors. As a means of internal validation, 10‐fold cross‐validation was used to evaluate the performance of the prediction models thoroughly. The added predictive value of incorporating candidate proteins into the Clinical Model (Composite Model) was also assessed. The discriminative capacity between different models was compared using the DeLong test, with a *P*‐value of less than 0.05 considered statistically significant. Time‐dependent ROC curves were constructed to assess the discriminative ability of each prediction model at clinically relevant time points: evaluations began two years after baseline and occurred every six months thereafter. Calibration assessment evaluated the agreement between predicted risk probabilities and observed outcome frequencies. Model calibration was assessed by visualizing the consistency between predicted and observed risk using line and bar plots. Goodness‐of‐fit tests further quantified this agreement by statistically evaluating how well the model predictions align with the actual outcomes. To investigate the clinical utility of candidate psoriasis prediction models, decision curve analysis was conducted using the “rmda” package. The net benefit metric integrates information on true positives (correctly identified patients who would benefit from intervention) and false positives (incorrectly identified patients who undergo unnecessary interventions), weighted according to the clinical consequences of these outcomes as reflected in the threshold probability. The threshold probability represents the minimum risk at which a decision‐maker would recommend intervention and implicitly encodes the relative harm of false‐positive versus false‐negative classifications. We assessed net benefit across a range of threshold probabilities from 0% to 25%, encompassing realistic clinical scenarios from aggressive screening (low thresholds) to conservative management (high thresholds). At each threshold, we calculated the net benefit for the Composite Model, Protein Model, and Clinical Model, as well as for two reference strategies: “treat all” (intervening in all patients regardless of predicted risk) and “treat none” (not intervening in any patients). The net benefit was calculated as the proportion of true positives minus the proportion of false positives, weighted by the odds of the threshold probability: *NB = (TP/n) – (FP/n) × [Pt/(1‐Pt)]*, where TP is the number of true positives, FP is the number of false positives, n is the total sample size, and Pt is the threshold probability. Decision curves were plotted with threshold probability on the x‐axis and net benefit on the y‐axis. A prediction model is considered clinically useful if its curve lies above both the “treat all” and “treat none” reference lines across clinically relevant threshold probabilities. The horizontal distance between a model's curve and the reference strategies quantifies the range of threshold probabilities over which the model provides clinical value. All decision curve analyses were based on apparent performance in the full cohort and employed 10‐fold cross‐validation.

**Step 5: In vivo validation of silencing PRSS8 and CDSN in psoriasis treatment**

### 2.19 Animals

BALB/c mice were purchased from the Guangdong Animal Experiment Center. The mice were maintained on a 12‐hour light/dark cycle with free access to food and water under standard pathogen‐free conditions. All animal experiments conducted in this study were approved by the Welfare and Ethical Committee for Experimental Animal Care of Southern Medical University. Mice were randomly divided into five groups (n = 5) before in vivo experiments.

To induce PsO‐like lesions, female BALB/c mice (6 weeks old) were treated with 62.5 mg of 5% imiquimod cream applied to the shaved dorsal skin once daily for six consecutive days. Control mice received an equivalent amount of Vaseline cream. Small interfering RNAs (siRNAs) targeting PRSS8 (si‐PRSS8) and CDSN (si‐CDSN) were synthesized by RIBOBIO Company. Si‐PRSS8 (2.5 nmol per mouse) and si‐CDSN (2.5 nmol per mouse) were injected subcutaneously on days 0, 2, and 4, with the same amount of siRNA negative control (si‐NC) administered to si‐NC group. The severity of skin lesion was assessed in a blinded manner by scoring erythema, thickness, and scaling on a scale from 0 to 4 (0: none; 1: slight; 2: moderate; 3: marked; 4: maximum), and the cumulative score was calculated.

### 2.20 Single Cell‑Type Expression Analysis

To evaluate the cell type‐specific expression of target genes implicated in psoriasis, we used single‐cell RNA‐sequence (scRNA‐seq) data from skin in healthy people and psoriatic patients (GSE162183). Seurat object was created by Seurat R package (version 5.0).

### 2.21 Histological Analysis

The dorsal skin from the mice was fixed in a 4% paraformaldehyde solution and embedded in paraffin. The tissue sections were then deparaffinized and rehydrated using a series of xylene and ethanol solutions. For histological evaluation, the sections were stained with hematoxylin and eosin (H&E).

### 2.22 Immunofluorescence Staining

After rehydration of mice, antigen retrieval was performed on the sections by heating them in a citrate buffer (pH 6.0) at 100 °C for 10 minutes. The subsequent procedures were conducted as previously described.^[^ ^32^ ^]^

### 2.23 Cell Culture and Experiments

The human keratinocyte HaCaT cells were grown and maintained in Dulbecco's modified Eagle's medium supplemented with 10% heat‐inactivated fetal bovine serum in a humidified chamber with 5% CO_2_ at 37 °C.

Starved HaCaT cells were stimulated by 10 µg mL^−1^ LPS and 10 ng mL^−1^ TNF‐α to mimic the inflammatory condition in psoriasis.^[^ ^33^ ^]^

### 2.24 Western Blotting

Skin tissues were lysed on ice using RIPA lysis buffer for 30 minutes. Equal amounts of protein extracted from different samples were separated by SDS‐polyacrylamide gel electrophoresis (SDS‐PAGE) and then transferred onto polyvinylidene fluoride (PVDF) membranes. The membranes were blocked with 5% BSA and immunoblotted overnight at 4 °C with primary antibodies against CDSN, PRSS8, and β‐actin (Proteintech). Following this, the membranes were incubated with an appropriate HRP‐conjugated secondary antibody for 1 hour at room temperature. The target proteins were detected using enhanced chemiluminescence reagent (Beyotime Biotechnology).

### 2.25 Quantitative Real‐Time PCR

Total RNA was extracted from skin biopsies using TRIzol, following the manufacturer's instructions and established protocols.^[^ ^34^ ^]^ The expression levels of the target genes were normalized to the expression of the β‐actin gene.

### 2.26 Flow Cytometry

Mice were euthanized, and the draining lymph nodes and spleen were harvested. The tissues were ground, and the resulting suspensions were filtered through a 70‐µm cell strainer to obtain single‐cell suspensions. These suspensions were then incubated with a cell stimulation cocktail (Thermo Fisher) for 6 hours and subsequently stained with fluorescent antibodies against CD45, CD3, and CD4 (eBioscience) for 30 minutes at 4 °C. After washing, cells were stained with Fixable Viability Dye, followed by cell fixation and permeabilization. Finally, the cells were stained for IL‐17 for 30 minutes at 4 °C. Flow cytometry was performed using a CytoFLEX analyzer (Beckman), and the data were analyzed with FlowJo software.

The information of the reagents used in this study was presented in Table S24 (Supporting Information).

## 3 Results

**Step 1: Effects of proteins on psoriatic disease based on gene‐level causal inference framework**

### 3.1 Screening Proteins with Potential Causal Associations with PsD

**Figure 2:**
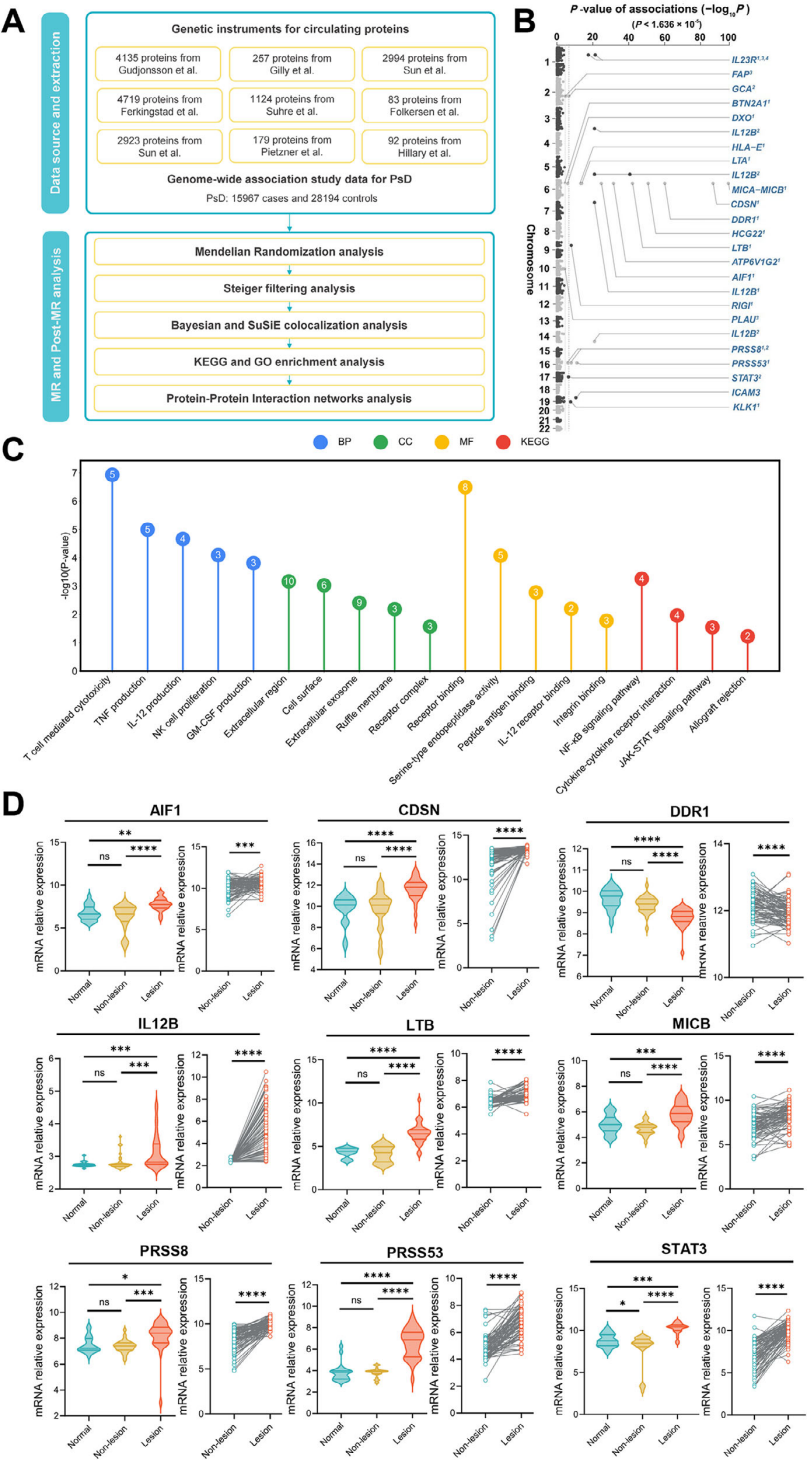
Screening PsD‐related proteins genetically and validating their expression transcriptionally. A) Flowchart of the gene‐level causal inference framework for evaluating the effects of plasma proteins on PsD. B) Manhattan plot for associations of genetically predicted protein with PsD in MR analysis. C) Lollipop plot of the enriched pathway in KEGG and GO analysis. D) Expression of mRNA in skin tissue from normal donors, lesional and non‐lesional PsD skin. 1 represents Sun et al. 2 represents Ferkingstad et al. 3 represents Gudjonsson et al. 4 represents Suhre et al. GO, gene ontology; KEGG, Kyoto Encyclopedia of Genes and Genomes; BP, biological process; CC, cellular component; MF, molecular function; AIF1, allograft inflammatory factor 1; CDSN, corneodesmosin; DDR1, epithelial discoidin domain‐containing receptor 1; IL12B, interleukin‐12 subunit beta; LTB, lymphotoxin‐beta; MICB, MHC class I polypeptide‐related sequence B; PRSS8, prostasin; PRSS53, serine protease 53; STAT3, signal transducer and activator of transcription 3.

To explore potentially causal associations between proteins and PsD, we conducted a comprehensive analysis integrating proteomic and genomic studies. The study design for this section is shown in **Figure** **2**A. Nine studies from Europe were integrated, resulting in the largest pQTLs dataset to date. We identified 9132 cis‐pQTLs for 3057 unique proteins to serve as instrumental variables for plasma proteins (Table S3, Supporting Information). At the threshold of Bonferroni‐corrected *P*‐value < 1.636 × 10^−^⁵, 22 genetically predicted proteins significantly associated with PsD were identified (Tables S4–S6, Supporting Information). Our study observed no significant heterogeneity and horizontal pleiotropy for the candidate proteins (Table S7 and S8, Supporting Information). Since genetically predicted associations between proteins and phenotypes may be explained by reverse causality, a directionality test namely Steiger filtering analysis was performed. The results of the Steiger test indicated that all MR‐identified associations exhibited the correct direction, demonstrating that the effects run from the proteins to PsD (Table S9, Supporting Information). The results of the bidirectional MR analysis showed that the influence of the disease on the proteins was not significant, further supporting the findings of the aforementioned test (Table S10, Supporting Information). Candidate causal proteins for PsD were highlighted in the Manhattan plot (Figure 2B). For example, IL‐23 is a cytokine produced by dendritic cells and other immune cells that promotes the development and activation of Th17 cells.^[^ ^35^ ^]^ The cytokines produced by these Th17 cells, such as IL‐17, play a crucial role in the pathogenesis of PsD.^[^ ^36^ ^]^ Monoclonal antibodies targeting IL‐23, such as ustekinumab and guselkumab, have been developed for the treatment of PsD.^[^ ^37^ ^]^ Additionally, STAT3 enhances the proliferation and activation of inflammatory cells in the inflammatory response associated with PsD, exacerbating the symptoms of the disease.^[^ ^35^ ^]^ Tofacitinib, an inhibitor of the JAK‐STAT pathway, concurrently inhibits the actions of multiple PsD‐related cytokines to alleviate the condition.^[^ ^37^ ^]^ These results demonstrate the crucial role of gene‐level identification of protein‐disease associations in drug development.

### 3.2 Colocalization and Functional Analysis of Candidate Causal Proteins

To further strengthen the evidence for both the causal and functional relevance of the candidate proteins to PsD, we performed colocalization, pathway, and interaction analyses. Colocalization analysis can determine whether the genetic associations between proteins and PsD are due to the same causal variant, thereby mitigating the potential confounding impact of linkage disequilibrium on the results. Our findings revealed strong colocalization support (PH_4_ > 0.8) for *Prolyl Endopeptidase (FAP), Interleukin‐23 Receptor (IL‐23R)*, *Serine Protease 53 (PRSS53)*, *Antiviral Innate Immune Response Receptor (RIGI)*, and *Signal Transducer and Activator of Transcription 3 (STAT3)* (Table S11, Supporting Information). The Gene Ontology (GO) analysis revealed significant enrichment in immune response and inflammation in candidate proteins, particularly T cell‐mediated cytotoxicity, tumor necrosis factor (TNF), and IL‐12 production, as well as the importance of cell surface and extracellular exosomes in intercellular communication and peptide antigen binding in immune recognition (Figure 2C; Table S12, Supporting Information). The Kyoto Encyclopaedia of Genes and Genomes (KEGG) pathway analysis emphasized the relevance of the NF‐κB and JAK‐STAT signaling pathways, further underscoring their roles in immune and inflammatory regulation (Figure 2C; Table S13, Supporting Information). The PPI network comprised 21 nodes with 41 edges (enrichment *P*‐value: 3.65 × 10^−^^13^), markedly surpassing the expected 10 edges, underscoring its biological significance (Figure S1, Supporting Information).

**Step 2: Expression of candidate proteins at the transcriptional level**

The GSE14905 and GSE30999 datasets from the GEO were used for the analysis in this section. In the skin tissues of 21 PsD patients compared to 29 healthy individuals, the relative mRNA expression levels of *CDSN*, *PRSS8*, A*llograft Inflammatory Factor 1 (AIF1)*, *Epithelial Discoidin Domain‐containing Receptor 1 (DDR1)*, *HLA class I Histocompatibility Antigen, Alpha Chain E (HLA‐E)*, *Interleukin‐12 Subunit Beta (IL12B)*, *Kallikrein‐1 (KLK1)*, *Lymphotoxin‐Beta (LTB)*, *MHC class I polypeptide‐related sequence A/B (MICA/MICB)*, *PRSS53*, and *STAT3* were significantly upregulated (Figure 2D; Figure S2, Supporting Information). Furthermore, significant differences in the mRNA levels of these proteins were observed between lesional and non‐lesional areas in 85 paired PsD patients (Figure 2D; Figure S2, Supporting Information). These results confirmed that the aforementioned proteins were indeed upregulated or downregulated at the transcriptional level.

**Step 3: Further evaluation based on population and protein levels**

Although the candidate proteins demonstrated potential causal associations with PsD at the genetic and transcriptional levels, further validation using population‐based data was necessary to confirm their risk stratification ability, discrimination capability, and relationships with disease specificity, activity, and severity.

### 3.3 Observational Evaluation in the UK Biobank Cohort

**Figure 3:**
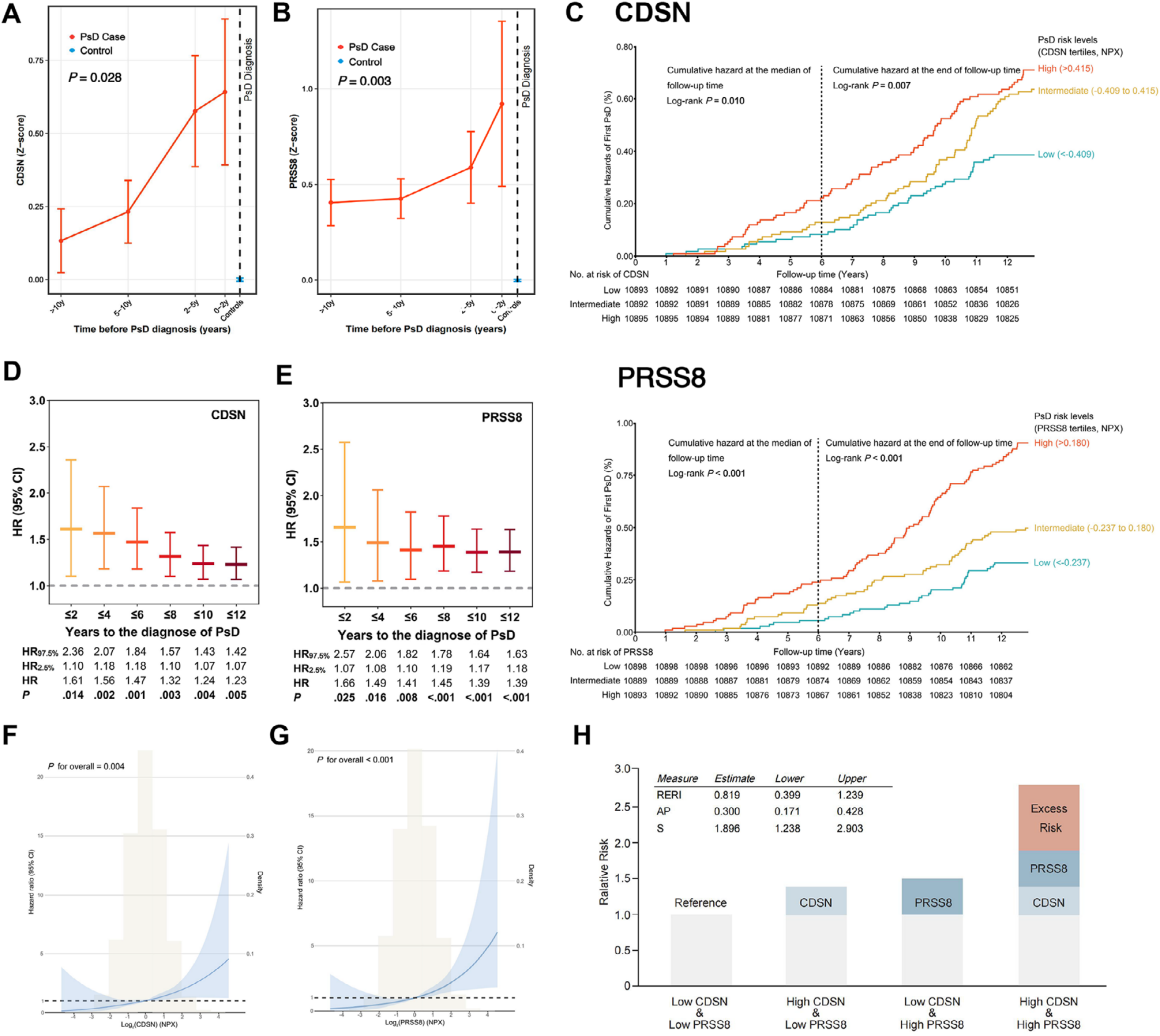
Associations of CDSN and PRSS8 with incident PsD risks in the UKB‐PPP. A,B) Comparison of baseline CDSN (A) and PRSS8 (B) levels between PsD cases and non‐cases during follow‐up. C) Kaplan‐Meier curves for PsD event timing across three strata of CDSN (Upper) and PRSS8 (Lower) levels. Raw, unadjusted protein levels were used. D,E) Adjusted HRs for PsD events from Cox proportional hazards regression analysis within 2, 4, 6, 8, 10, and 12 years from the baseline. F,G) The dose‐response relationship between CDSN (F), PRSS8 (G) and incident PsD using restricted cubic spline analysis. H) Additive interaction of CDSN and PRSS8 on incident PsD: RERI as Relative Excess Risk, AP as Attributable Proportion, S as Synergy index. NPX, normalized protein expression; SE, standard error; HR, hazard ratio; CDSN, corneodesmosin; PRSS8, prostasin; PsD, psoriatic disease.

Detailed baseline information is provided in Table S14 (Supporting Information). In summary, 192 participants developed incident PsD during the follow‐up. Compared to those without PsD, participants with PsD were older (mean age 60 vs 57 years), had a higher BMI (median 28.65 vs 27.47 kg m^−^^2^), and had a higher proportion of males (53.12% versus 45.11%). Additionally, we observed that baseline levels of five proteins were significantly higher in the PsD group compared to the non‐PsD group (Table S14, Supporting Information). We also found that the protein concentrations of CDSN and PRSS8 gradually increased as the diagnosis approached, indicating a significant temporal trend (Jonckheere‐Terpstra test, *P* < 0.05) (**Figure** **3**A,B).

To identify the potential of candidate proteins as predictive biomarkers, we conducted a longitudinal analysis of their temporal relationship with PsD incidence. The Log‐rank test indicated significant differences in PsD incidence among three risk groups over six and twelve years of follow‐up for nine proteins (all *P*‐values < 0.05, Figure 3C; Figure S3, Supporting Information). Kaplan‐Meier curves illustrated that patients in the higher risk categories stratified by the tertile of CDSN, PRSS8, and Decapping and Exoribonuclease Protein (DXO) exhibited a higher risk of PsD events (Figure 3C; Figure S3, Supporting Information). Over the 12‐year follow‐up, we found that each standard deviation (SD) increase in the expression levels of CDSN, PRSS8, DDR1, DXO, and PRSS53 was associated with a 23%, 39%, 26%, 22%, and 19% increase in PsD risk, respectively (Figure 3D,E; Table S15, Supporting Information). The stratified subsets included cases diagnosed at 2, 4, 6, 8, 10, and 12 years from baseline. The results showed that only CDSN, PRSS8, and DXO exhibited consistent associations between increased concentration and elevated risk of PsD across all time intervals (Figure 3D,E; Table S15, Supporting Information). To further assess the discriminative capacity of the candidate proteins for the disease, we calculated the area under the receiver operating characteristic curve (AUC) for PsD events. The results revealed that CDSN, PRSS8, and LTA exhibited discriminatory power, with AUCs of 0.64, 0.65, and 0.66, respectively (Table S16, Supporting Information).

Based on the above analyses, CDSN and PRSS8 were robustly validated across genetic, transcriptional, and population‐level assessments, emerging as highly promising candidates (Table S17, Supporting Information). These two proteins demonstrated substantial potential as candidate therapeutic targets and predictive biomarkers for PsD. Furthermore, we aimed to investigate the dose‐response relationship between CDSN, PRSS8, and the risk of incident PsD. The restricted cubic spline indicated that there was indeed a dose‐response relationship; as the protein levels increased, the risk of PsD significantly rose (both *P*‐value for overall < 0.005, Figure 3F,G). An additive interaction effect on PsD between CDSN and PRSS8 was also observed. Compared to the group with low levels of CDSN and PRSS8, the group with high levels of CDSN and PRSS8 had nearly three times higher risk of incident PsD, with an excess risk of 0.819 (0.399, 1.239), and the attributable proportion of excess risk reached 30% (Figure 3H).

### 3.4 Additional Evaluation of CDSN and PRSS8 as Candidate Biomarkers for Incident PsD

Given the long follow‐up duration, it is necessary to perform a competing risk analysis. The Fine‐Gray subdistribution hazard model confirmed that CDSN (sHR = 1.21, 95% CI: 1.06‐1.40, *P*‐value = 0.006) and PRSS8 (sHR = 1.40, 95% CI: 1.19‐1.65, *P*‐value < 0.001) remained significantly associated with the incidence of PsD after accounting for death as a competing risk and adjusting for age, sex, BMI, and batch effects. Due to the relatively low incidence of PsD, conducting a propensity score matching (PSM) analysis as a sensitivity check would provide further validation of the stability of the associations between candidate biomarker levels and the risk of PsD. A 1:1 propensity score matching method was employed to compare populations with and without PsD, with adjustments made for confounding variables such as age, sex, and BMI. Following propensity score matching, the baseline characteristics were balanced between the two groups (Table S18, Supporting Information). Subsequent analyses demonstrated that CDSN was associated with an increased risk of incident PsD (HR = 1.17, 95% CI: 1.01‐1.35, *P*‐value = 0.034), while PRSS8 was similarly associated with a heightened risk (HR = 1.21, 95% CI: 1.04‐1.40, *P*‐value = 0.012). Furthermore, other potential confounders such as medication use, comorbidities, and genetic background may influence the relationship between biomarkers and disease. Therefore, we performed additional adjustments for participants' medication use (including medications used for treating cholesterol, blood pressure, diabetes, and exogenous hormone therapy), self‐rated health as an indicator of comorbidity burden, polygenic risk scores (PRS) for PsD, and socioeconomic factors (measured by the Townsend Deprivation Index) (Table S19, Supporting Information). The results indicated that the primary associations remained significant following further adjustment. Specifically, the HR for CDSN associated with the risk of incident PsD was 1.21 (95% CI: 1.05‐1.40, *P*‐value = 0.009), while the HR for PRSS8 was 1.38 (95% CI: 1.16‐1.63, *P*‐value < 0.001).

To further assess the specificity of the associations between CDSN, PRSS8, and PsD, we conducted a literature review to identify conditions that need to be differentiated from PsD as follows: atopic dermatitis (L20), seborrheic dermatitis (L21), lichen simplex chronicus (L28), pruritus (L29), pityriasis rosea (L42), and lichen planus (L43). CDSN and PRSS8 showed no significant associations with the incidence of these conditions, demonstrating good specificity (Figure S4, Supporting Information).

**Figure 4:**
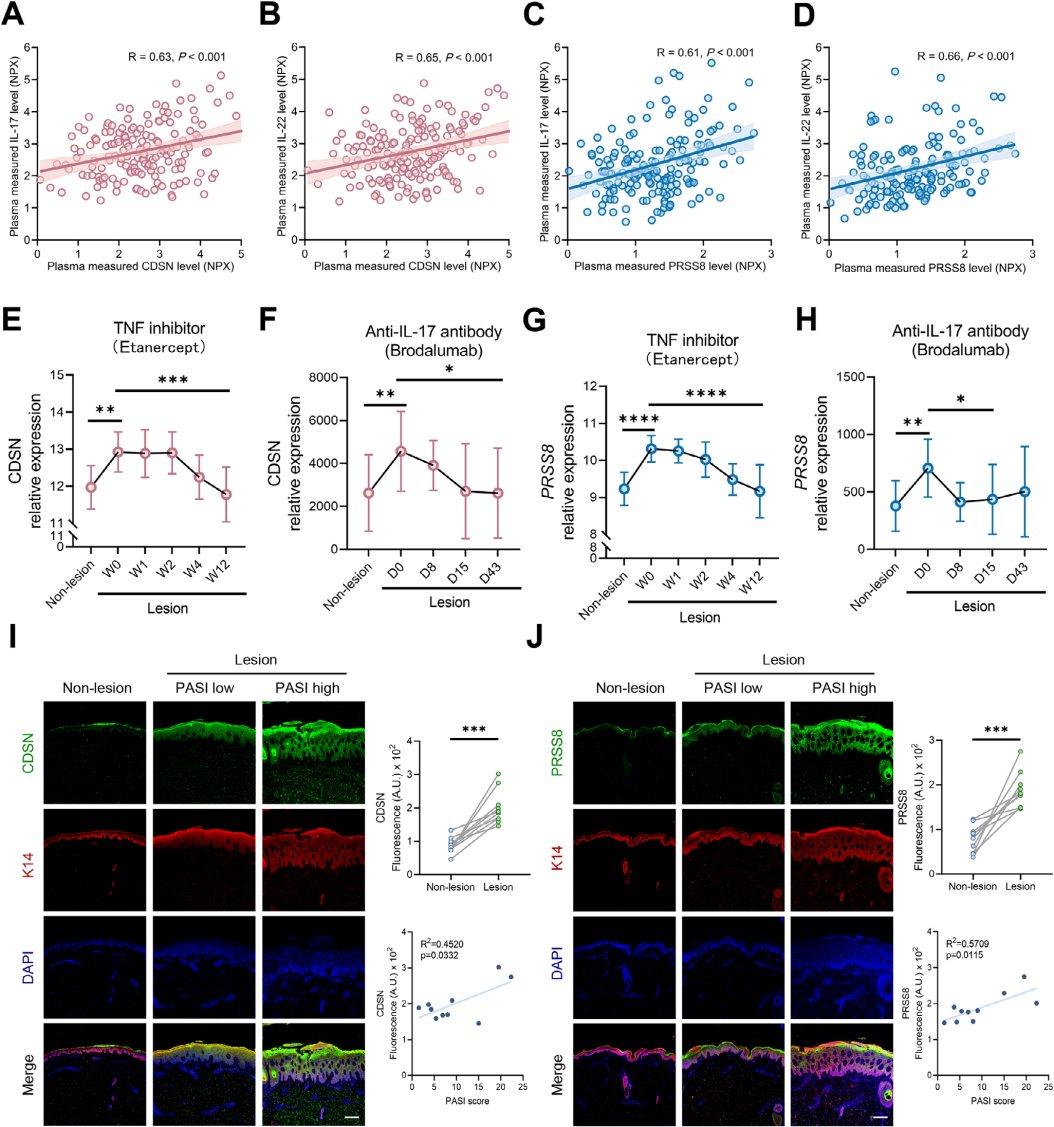
Correlation of CDSN and PRSS8 with disease activity and severity in PsD. A,B) Pearson correlation of CDSN with IL‐17 and IL‐22 levels in PsD patients with joint damage (n = 185). C,D) Pearson correlation of PRSS8 with IL‐17 and IL‐22 levels in PsD patients with joint damage (n = 167). E,F) The expresion of CDSN in PsD patients after administration of TNF‐α (E) or IL‐17 inhibitor (F) through analysis of GEO database. G,H) The expresion of PRSS8 in PsD patients after administration of TNF‐α (G) or IL‐17 inhibitor (H). I,J) The expression of CDSN (I) or PRSS8 (J) and K14 in non‐lesional and lesional skin of psoriatic patients. Scale bar = 100 µm. The correlation of CDSN and PRSS8 with PASI score was also presented. CDSN, corneodesmosin; PRSS8, prostasin; IL‐17, interleukin‐17; IL‐22, interleukin‐22; TNF, tumor necrosis factor; PASI, psoriasis area and severity index; PsD, psoriatic disease.

Studies have demonstrated that the circulating levels of IL‐17 and IL‐22 are elevated in PsD patients compared to healthy controls, and are closely associated with disease activity. Our findings showed significant correlations of CDSN/PRSS8 with IL‐17/IL‐22 in PsD patients, suggesting that CDSN and PRSS8 are implicated in disease activity (all *P*‐value < 0.001, the data came from the UK Biobank, **Figure** **4**A–D). Moreover, both CDSN and PRSS8 expression in skin of PsD patients declined after the application of TNF‐α inhibitor (Etanercept) or IL‐17 inhibitor (Brodalumab) in clinical practice, which further confirmed that CDSN and PRSS8 are closely associated with PsD activity (the data came from GSE11903 and GSE53552 in GEO, Figure 4E–H). Additionally, the expression of PRSS8 or CDSN not only significantly increased in lesional skin compared with non‐lesional skin but also positively correlated with patients’ PASI score (a recommended and widely accepted measure for assessing the severity of PsD in clinical practice) (the data were obtained from PsD patients enrolled by our team, Figure 4I,J). These results suggest that CDSN and PRSS8 may be important candidate biomarkers for PsD.

**Step 4: Development and evaluation of the PsD prediction model**

### 3.5 Selection of Clinical Predictors and Assessment of Predictive Performance

**Figure 5:**
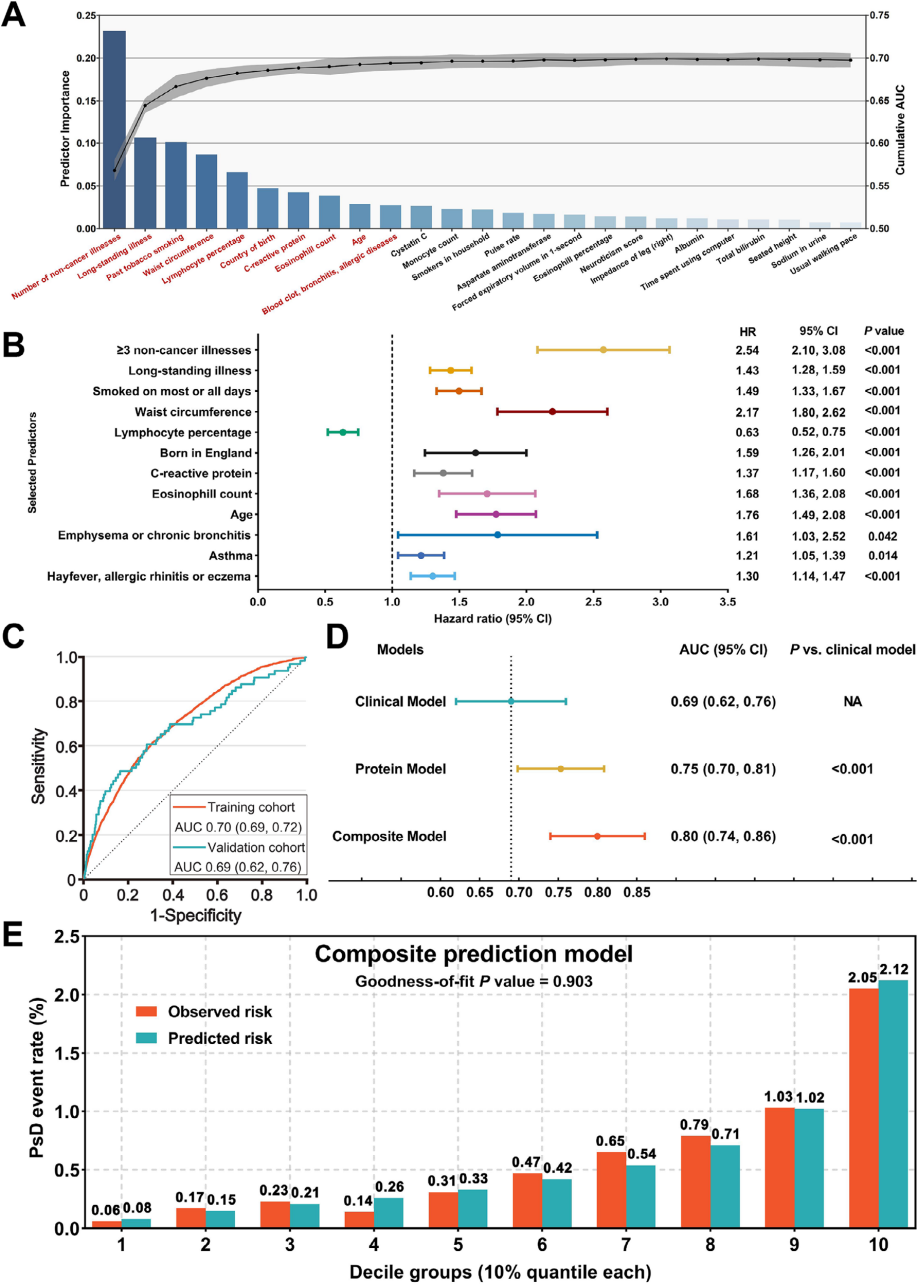
Development and validation of predictive models. A) Sequential forward selection from pre‐selected candidate clinical predictors. B) Associations between top‐10 clinical predictors and PsD from multivariable Cox proportional hazards regression analysis. C) Receiver operating characteristic (ROC) curves of the clinical prediction model in the training and validation cohort. D) Discrimination and reclassification improvement by CDSN and PRSS8 for PsD risk prediction. E) Calibration plots of the composite prediction model on PsD. AUC, the area under the ROC curve; HR, hazard ratio.

This section aims to validate the concept that candidate proteins (CDSN and PRSS8) can enhance the predictive model of PsD. However, given the lack of a reliable predictive model for PsD, we developed a reference model based on clinical factors. The non‐UKB‐PPP cohort (training cohort) comprised 353578 participants, including 1697 diagnosed with PsD. Meanwhile, the UKB‐PPP cohort (validation cohort) included 29432 participants, with 172 diagnosed with PsD (**Table** **1**). Among the 289 candidate clinical predictors (Table S22, Supporting Information), the initial selection process identified 50 candidates, which were then subjected to hierarchical clustering to eliminate multicollinearity (Figure S5A,B, Supporting Information). This resulted in the final selection of 25 predictors, ranked according to their importance for the prediction task, as shown in the bar chart (**Figure** **5**A). Ultimately, we selected the top 10 variables as the final predictors for the development of the LightGBM model.

**Table 1:** The baseline information of UKB participants included in the development of predictive models (used in Step 4).

	Training cohort [n = 353578]			Validation cohort [n = 29342]		
Variables	PsD events (n = 1697)	Non‐PsD events (n = 351881)	*P‐*value	PsD events (n = 172)	Non‐PsD events (n = 29170)	*P‐*value
Age (years)	58.77 ± 7.58	56.61 ± 8.05	**<0.001**	60.05 ± 7.19	56.94 ± 8.19	**<0.001**
Female sex	811 (47.8)	192496 (54.7)	**<0.001**	79 (45.9)	15921 (54.6)	**0.026**
BMI (kg/m^2^)	29.14 ± 5.49	27.38 ± 4.71	**<0.001**	28.36 ± 5.06	27.44 ± 4.77	**0.012**
Ethnicity (White)	1571 (92.6)	321467 (91.4)	**0.046**	158 (91.9)	26380 (90.4)	0.770
Number of non‐cancer illnesses	2.85 ± 2.27	1.80 ± 1.82	**<0.001**	2.89 ± 2.11	1.91 ± 1.89	**<0.001**
Long‐standing illness	878 (51.7)	108903 (30.9)	**<0.001**	88 (51.2)	9830 (33.7)	**<0.001**
Smoking frequency			**<0.001**			**<0.001**
Most or all days	678 (40.0)	91722 (26.1)		77 (44.8)	7768 (26.6)	
Occasionally	234 (13.8)	50242 (14.3)		24 (14.0)	4124 (14.1)	
Sometimes or never	785 (46.2)	209917 (59.6)		71 (41.2)	17278 (59.3)	
Waist circumference (cm)	95.70 ± 14.46	90.04 ± 13.36	**<0.001**	93.88 ± 14.69	90.17 ± 13.36	**<0.001**
Lymphocyte percentage (%)	27.43 ± 7.94	29.00 ± 7.46	**<0.001**	27.59 ± 8.00	28.97 ± 7.64	**0.018**
Country of birth			**<0.001**			**0.030**
England	1466 (86.4)	275998 (78.4)		150 (87.2)	22470 (77.0)	
Scotland	84 (4.9)	28316 (8.0)		7 (4.1)	2390 (8.2)	
Wales	45 (2.7)	15923 (4.5)		3 (1.7)	1394 (4.8)	
Ireland	28 (1.6)	5476 (1.7)		3 (1.7)	459 (1.6)	
Elsewhere	74 (4.4)	26168 (7.4)		9 (5.3)	2457 (8.4)	
C‐reactive protein (mg/L)	3.57 ± 5.73	2.46 ± 4.19	**<0.001**	3.75 ± 7.33	2.52 ± 4.21	**<0.001**
Eosinophill count (10^9^/L)	0.20 ± 0.16	0.17 ± 0.14	**<0.001**	0.17 ± 0.14	0.17 ± 0.14	0.810
Blood clot	72 (4.2)	8382 (2.4)	**<0.001**	6 (3.5)	781 (2.7)	**0.010**
Emphysema or chronic bronchitis	40 (2.4)	4527 (1.3)	**<0.001**	6 (3.5)	403 (1.4)	**<0.001**
Asthma	270 (15.9)	37727 (10.7)	**0.032**	15 (8.7)	3095 (10.6)	**0.048**
Hayfever, allergic rhinitis or eczema	334 (19.7)	62760 (17.8)	**0.044**	37 (21.5)	5186 (17.8)	**0.031**

Data were presented as numbers (percentages) or mean value ± standard deviation (SD). Categorical variables were analyzed using a two‐sided χ^2^ test. Continuous data were analyzed using a two‐sided t‐test. *P*‐value < 0.05 was considered as significant. UKB, UK Biobank; PsD, psoriatic disease; BMI, body mass index.

To better visualize the contribution of each predictor to the LightGBM model, we generated SHAP plots (Figure S5C, Supporting Information). The figure could be interpreted from two aspects: The horizontal range visually reflected the overall predictive power of each predictor (CDSN, PRSS8, and the number of non‐cancer diseases had the widest range and strongest predictive power); the values on the x‐axis and their trend direction reflected the specific effects (for example, participants with high PRSS8, marked in red, tended to be on the right side of the high PsD risk, while those with low PRSS8, marked in blue, clustered on the left side of the low risk). We further conducted multivariable Cox regression with clinical predictors as the exposures and PsD as the outcome. The results demonstrated that all ten clinical variables included in the model were significantly associated with PsD. Aside from the lymphocyte percentage, which was negatively associated with PsD incidence, all other variables were positively correlated with PsD incidence (Figure 5B). The above analysis indicated that the clinical predictors included in our model were all effective and non‐redundant.

### 3.6 Training and Validation of PsD Prediction Models

The PsD prediction model was constructed using the LightGBM, and the model's discriminatory ability was evaluated using AUC. Notably, the prediction span in this study extends up to 12 years. According to Figure 5C, the Clinical Model had an average AUC of 0.70 (0.69, 0.72) in the training cohort and an AUC of 0.69 (0.62, 0.76) in the validation cohort. As a means of internal validation, 10‐fold cross‐validation was used to evaluate the performance of the prediction models thoroughly. Subsequently, the Protein Model, including CDSN and PRSS8, achieved a corrected AUC of 0.75 (0.70, 0.81) in the UKB‐PPP cohort (Figure 5D). The Composite Model, which incorporated clinical predictors along with the two proteins, attained a corrected AUC of 0.80 (0.74, 0.86) for predicting PsD over 12 years. In the DeLong test, both the Protein Model and the Composite Model demonstrated significantly higher discriminative ability compared to the reference model (Clinical Model) (Figure 5D). The calibration was evaluated using the Hosmer‐Lemeshow goodness‐of‐fit test, with a *P*‐value greater than 0.05 indicating an adequate fit of the model. For the Composite Model, the calibration plot for PsD events showed excellent fit (*P*‐value = 0.903), with predicted probabilities closely matching observed proportions across all deciles (Figure 5E; Figure S8, Supporting Information). In addition, we have included time‐dependent ROC curves, which illustrate that when the prediction time frame is limited to six years, the AUC value of the Composite Model reaches 0.91 (Figure S6, Supporting Information). We have supplemented our analysis with the decision curve analysis results. At clinically relevant thresholds (5‐10%), the Composite Model achieved net benefits of 0.030‐0.035, equivalent to the Protein Model, but higher than the Clinical Model (0.008‐0.003) (Figure S7, Supporting Information). The Composite Model and the Protein Model maintained clinical utility across the widest range of thresholds, supporting their use for risk stratification in diverse clinical settings. In summary, we successfully developed a predictive model based on proteins and clinical predictors, and demonstrated a significant improvement in model performance with the inclusion of the proteins. This further confirms the clinical application potential of CDSN and PRSS8 as predictive biomarkers.

**Step 5: In vivo validation of silencing *CDSN* and *PRSS8* in PsD treatment**

### 3.7 Targeting CDSN and PRSS8 Therapy Ameliorates PsD Progression

Previous studies reported that abnormal high expression of CDSN and PRSS8 are closely associated with skin dysfunction and inflammation, respectively.^[^ ^38^, ^39^ ^]^ Additionally, in this study, CDSN and PRSS8 demonstrate potential as therapeutic targets in PsD, with further showing an additive interaction effect on the risk of PsD. Therefore, we are interested in experimentally validating whether targeting the local skin expression of *CDSN* and *PRSS8*, both individually and in combination, can exert therapeutic effects.

**Figure 6:**
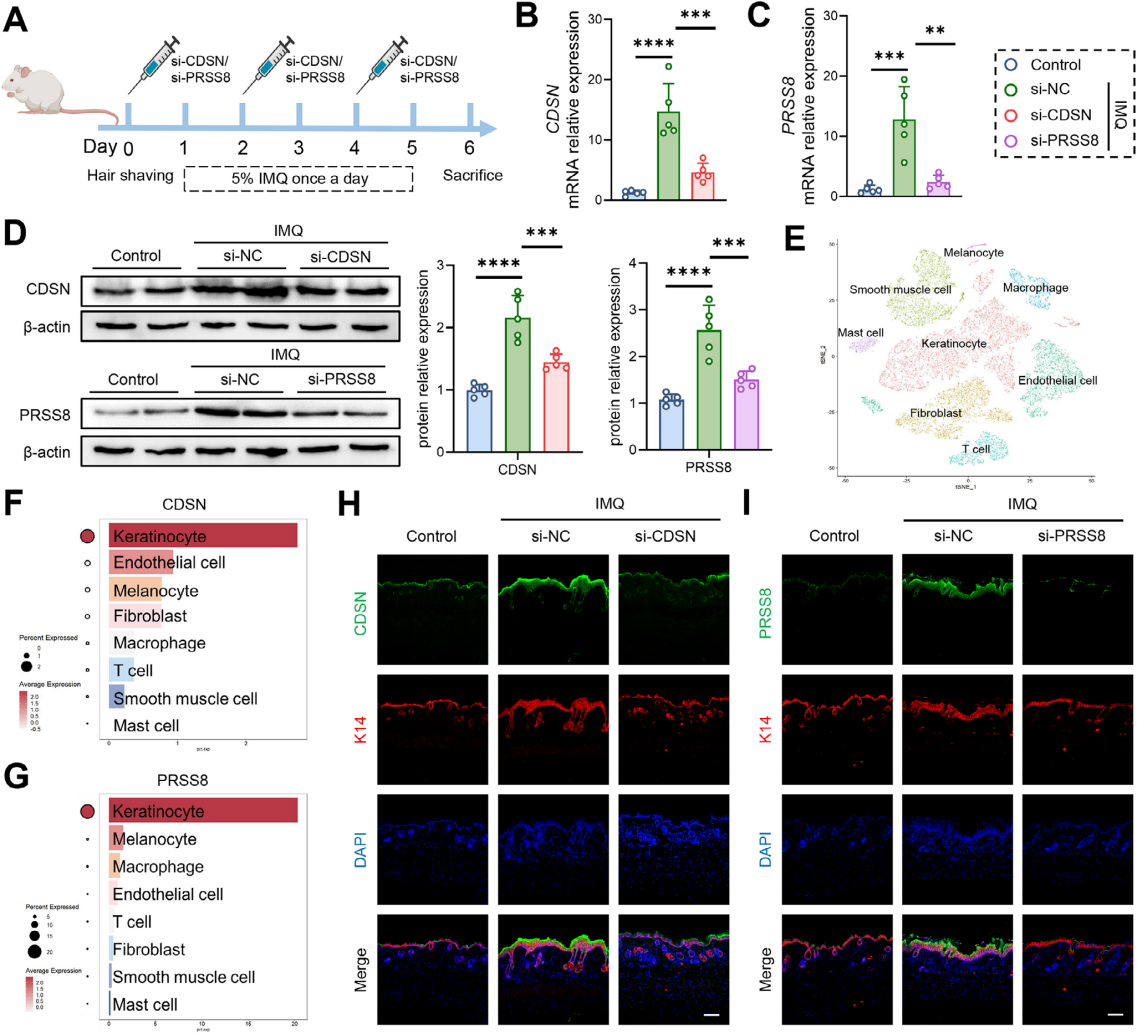
CDSN and PRSS8 exhibited increased expression in keratinocytes of PsD‐like skin lesions. A)The scheme of BALBc mice received IMQ with or without si‐PRSS8 or si‐CDSN treatment. B,C) The mRNA levels of CDSN (B) and PRSS8 (C) in the skin tissues from mice measured by RT‐qPCR. D) The protein levels of CDSN and PRSS8 in the skin tissues from mice measured by Western blotting. E–G) The distribution of CDSN and PRSS8 in PsD patients by single‐cell sequencing. H,I) Representative immunofluorescence staining of skin sections for CDSN (H) or PRSS8 (I) and K14 expression. PRSS8, prostasin; CDSN, corneodesmosin; IMQ, imiquimod.

In vivo siRNA against *CDSN* and *PRSS8* were administrated subcutaneously during mice PsD model (**Figure** **6**A). Surprisingly, we found that both *CDSN* and *PRSS8* expression increased in IMQ‐induced PsD‐like skin lesions, while were inhibited after the application of in vivo siRNA (Figure 6B–D). By the analysis of single‐cell sequencing of PsD patients, *CDSN* and *PRSS8* were both mainly expressed in the keratinocyte population (Figure 6E–G), which was corresponded with immunofluorescence in mice skin (Figure 6H,I).

**Figure 7:**
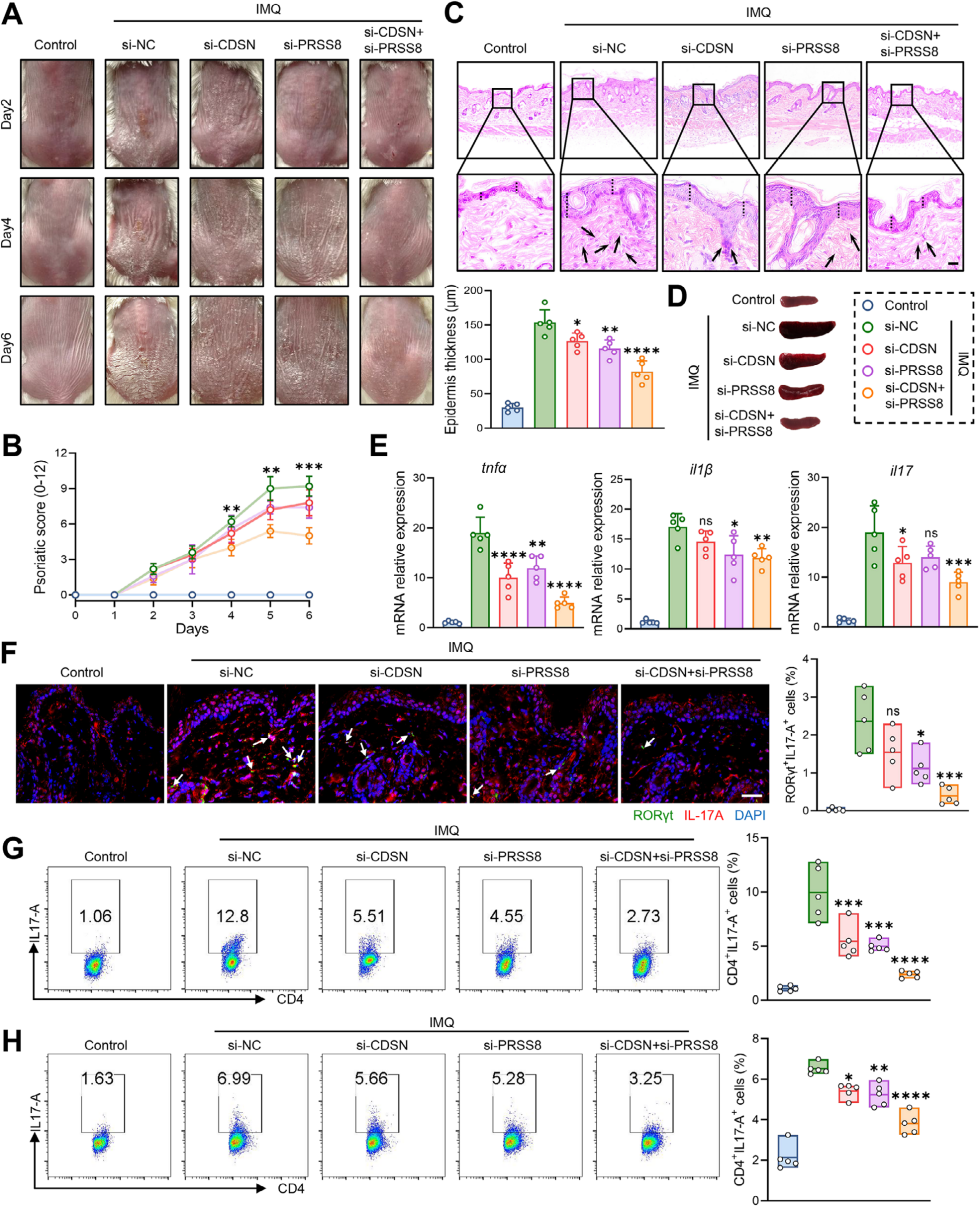
si‐CDSN plus PRSS8 significantly suppressed abnormal skin hyperplasia and excessive immune response. A) Macroscopic phenotypical representation of PsD‐like lesions in the mice at the indicated time point after IMQ stimulation. B) The cumulative score (erythema, thickness plus scaling) was scored daily on a scale from 0 to 12. C) H&E staining of back skin sections from mice, scale bar = 100 µm. D) The photo of mice spleen. E) Relative mRNA expression levels of the indicated pro‐inflammatory cytokines in the skin lesions. F) The frequency of Th17 cell in mice skin via immunofluorescence, scale bar = 50 µm. G) The frequency of Th17 cell in mice spleen via flow cytometry. H) The frequency of Th17 cell in mice lymph nodes via flow cytometry. CDSN, corneodesmosin; PRSS8, prostasin; IMQ, imiquimod.

Compared with the si‐NC group, mice that received either single si‐CDSN or si‐PRSS8 exhibited milder psoriatic pathogenesis (including scaling, thickness, and erythema), especially mice received both si‐CDSN and si‐PRSS8 treatment (**Figure** **7**A,B). Accordingly, H&E staining (Figure 7C) and spleen (Figure 7D) also indicated that single or combined si‐PRSS8 plus si‐CDSN significantly suppressed abnormal skin hyperplasia and excessive immune response during PsD development. As hyperinflammation was centered in PsD initiation and progression, the expression of inflammatory cytokines (including TNF‐α, IL‐1β, and IL‐17) as well as the Th17 population in local skin were remarkably lower in the si‐PRSS8 plus si‐CDSN group in comparison with the si‐NC (Figure 7E,F). Accordingly, the Th17 flow cytometry on spleen and lymph nodes also validated si‐RNA targeting CDSN and PRSS8 alleviated systemic immune response of PsD (Figure 7G,H). Besides, in vitro experiments also validated that knockdown of CDSN and PRSS8 could effectively inhibit the inflammatory condition of keratinocytes (Figure S9, Supporting Information). In summary, inhibition of CDSN and PRSS8 in the local skin area appears to be a promising and effective therapy against PsD.

## 4 Discussion

Currently, there is no cure for PsD, and no specific biomarkers are available for early risk prediction.^[^ ^1^, ^2^ ^]^ Using a population‐based gene‐level causal inference framework and expression level validation, coupled with a longitudinal cohort study, we identified CDSN and PRSS8 as novel genes associated with PsD. Additionally, we employed data‐driven and machine‐learning approaches to comprehensively screen clinical predictors for PsD. Promisingly, our machine learning model based on clinical and protein biomarkers achieved 80% accuracy in predicting PsD more than 12 years. Notably, silencing CDSN and PRSS8 in mice effectively reduced skin lesions and systemic inflammation, highlighting their potential as novel therapeutic targets for PsD.

To our knowledge, this is the first study to identify CDSN and PRSS8 as both predictive biomarkers and therapeutic targets for psoriatic disease using an integrated approach. CDSN, an adhesive protein, maintains cohesion and intercellular integrity in the skin.^[^ ^40^ ^]^ The expression of CDSN is increased and more widely distributed in psoriatic lesions compared to normal skin.^[^ ^38^ ^]^ Its gene expression is also positively associated with PsD risk.^[^ ^41^ ^]^ It is noteworthy that some current studies found complete CDSN deletion in skin could lead to peeling skin disease,^[^ ^42^, ^43^ ^]^ indicating normal CDSN expression is vital to skin physiological function, while complete loss or abnormal high of CDSN is detrimental. PRSS8 is a serine protease expressed in the epithelium of various organs, such as the prostate, skin, and so on.^[^ ^39^ ^]^ Previous studies proved that PRSS8 overexpression in the skin severely impaired the epidermal barrier function and provoked excessive inflammation.^[^ ^44^, ^45^ ^]^ Emerging evidence demonstrated that epidermal barrier dysfunction contributes greatly to initiating and perpetuating skin inflammation in the pathogenesis of PsD.^[^ ^46^ ^]^ Based on public scRNA‐seq data and immunofluorescence, CDSN and PRSS8 were both enriched in keratinocytes and the expression levels of these two molecules increased during PsD, while the expression significantly decreased after biological agents (TNF‐α or IL‐17 antibody) administration. Consistently, we found that CDSN and PRSS8 are positively correlated with PASI scores, IL‐17 and IL‐22 levels in PsD patients, suggesting that CDSN and PRSS8 play essential roles in the systematic inflammation of PsD. In summary, abnormally high expression of CDSN and PRSS8 may trigger the skin lesion and contribute to systemic as well as skin local inflammation‐related complications, ultimately leading to PsD.

Apart from CDSN and PRSS8, our study identified more than 20 proteins associated with PsD. Among those, IL23R, IL12B, and STAT3 have been confirmed as key proteins involved in the activation mechanisms of PsD.^[^ ^47^ ^]^ Iremovab targets the IL‐12 and IL‐23 axis, reducing the release of cytokines by T cells, thereby suppressing the abnormal immune response in PsD.^[^ ^48^ ^]^ Tofacitinib, an inhibitor of the JAK‐STAT pathway, simultaneously inhibits the action of multiple PsD‐related cytokines to alleviate PsD.^[^ ^49^ ^]^ Additionally, NF‐κB is considered a crucial factor in the pathogenesis of PsD.^[^ ^50^ ^]^ Proteins associated with the NF‐κB pathway, such as lymphotoxin‐alpha (LTA), LTB, and RIGI, may play significant roles in regulating keratinocyte proliferation and inflammatory infiltration.^[^ ^50^ ^]^ Proteins like urokinase‐type plasminogen activator (PLAU), Kallikrein‐1 (KLK1), intercellular adhesion molecule 3 (ICAM3), DDR1, and FAP have not yet been implicated in the pathogenesis of PsD. These proteins are considered druggable genetically and have been identified as drug targets for other diseases.^[^ ^51^, ^52^ ^]^ Cumulatively, our study has identified several proteins with potential as therapeutic targets for PsD. However, their specific roles in the pathogenesis of PsD warrant further investigation.

International guideline indicates that even a delay of just six months in diagnosing PsD is linked to significantly reduced treatment response.^[^ ^53^ ^]^ In contrast, early intervention with immune‐modulating or anti‐inflammatory drugs substantially improves both clinical and radiographic outcomes, highlighting the concept of a window of opportunity for therapeutic interventions.^[^ ^54^ ^]^ Therefore, we employed machine learning algorithms to establish the first PsD predictive model based on clinical and protein predictors for early prevention. Among the top ten clinical predictors, chronic diseases, smoking, obesity (waist circumference), age, and various allergic conditions have been confirmed as independent risk factors for PsD in previous studies.^[^ ^1^, ^2^ ^]^ Additionally, PsD was observed to have a higher incidence in developed regions, such as England.^[^ ^55^ ^]^ We also found that specific blood markers, such as lymphocyte percentage, C‐reactive protein, and eosinophil count, are independently associated with PsD. These findings offered new insights into the risk factors for PsD. Furthermore, we provided proof‐of‐concept that protein biomarkers have the potential to improve prediction models for PsD. In our study, the protein prediction model achieved an AUC of 0.75. The inclusion of protein biomarkers significantly enhanced the performance of the clinical model, raising the AUC from 0.69 to 0.80. Furthermore, the diagnostic challenge of skin diseases lies in differentiation, as many skin conditions have similar clinical manifestations.^[^ ^56^ ^]^ Therefore, biomarkers should also demonstrate good value in differential diagnosis. Based on authoritative guidelines, we examined the association of the biomarkers with seven skin diseases that need to be differentiated from PsD.^[^ ^37^ ^]^ The results indicated no association between them, highlighting the specificity of CDSN and PRSS8 as biomarkers for PsD.

It is important to note that a key limitation of the clinical model is that it primarily consists of factors that are difficult to modify, such as medical and lifestyle history.^[^ ^57^ ^]^ In contrast, plasma protein levels can be changed readily. Therefore, clinicians can use the protein model not only to identify individuals at higher risk for PsD but also to monitor their responses to changes in lifestyle and medication.^[^ ^58^, ^59^ ^]^ Our decision curve analysis demonstrates that the Composite Model provides substantial clinical value across a wide range of decision thresholds. The crossover point at 1.5% threshold probability indicates that clinicians with even modest risk aversion would benefit from using the Composite Model rather than universal screening. Importantly, the incremental net benefit of the Composite Model over the Protein Model (0.003‐0.005 across the 5–15% threshold range) should be weighed against the additional complexity and cost of incorporating clinical variables. In settings where biomarker assays are readily available but clinical assessment resources are limited, the Protein Model alone may offer an acceptable balance between performance and feasibility. However, there remains a need to identify more effective biomarkers to achieve more accurate early detection of PsD.

Notably, the therapeutic potential of our findings is further amplified by recent advancements in siRNA‐based therapies.^[^ ^60^ ^]^ Numerous siRNA drugs have entered clinical trials, demonstrating remarkable efficacy.^[^ ^61^ ^]^ The skin, being the largest and most accessible human organ, offers an ideal target for local siRNA delivery.^[^ ^62^ ^]^ SiRNA therapies offer distinct advantages over conventional drugs, including the ability to silence targets previously considered undruggable, a shorter development timeline, and extended clinical durability,^[^ ^63^ ^]^ maintaining targeted genes expression in relatively low level which prevents secondary skin dysfunction or diseases, such as peeling skin syndrome induced by complete loss of CDSN. These attributes highlight the potential of siRNA as a transformative approach in treating various conditions, including PsD. In this study, si‐CDSN and si‐PRSS8 were applied during PsD development, in which this intervention successfully inhibited the expression of CDSN and PRSS8 in the skin tissues. Remarkably, mice received si‐PRSS8 or si‐CDSN exhibited thinner skin and deceased psoriatic lesions, with controlled inflammatory levels as well as restricted IL‐17 immune response both in local skin and whole body. Together, inhibition of CDSN and PRSS8 by siRNA within the skin microenvironment could be a promising therapy for patients suffering from PsD. As molecular design and extrahepatic delivery platforms continue to progress, there will be a significant expansion in the application of siRNA drugs.^[^ ^64^ ^]^

This study had some limitations. Firstly, our study population is of European descent, so the applicability of these findings to other ethnic groups remains uncertain. Therefore, further external validation of the prediction model is needed in non‐European populations or independent real‐world datasets to ensure its broader applicability. Secondly, while we assessed the performance of the prediction models using cross‐validation, ideally, external validation on independent datasets is required to confirm the generalizability and robustness of the models. Thirdly, machine learning algorithms for predictor selection are data‐driven, which may introduce potential biases. However, we further illustrated their real‐world implications using SHAP plots and epidemiological methods.

In conclusion, we established a multi‐dimensional discovery pipeline integrating gene‐level causal inference framework and transcriptomic analyses with longitudinal clinical data to identify candidate causal proteins as both biomarkers and drug targets for PsD. Importantly, we translated these findings into clinically actionable tools through development of risk prediction models and experimental validation of CDSN and PRSS8 as therapeutic targets in vivo and in vitro. This work advances precision medicine approaches for PsD by enabling early risk identification and providing genetically supported targets for therapeutic development, with implications for disease prevention and improved clinical management.

## Conflict of Interest

The authors declare no conflict of interest.

## Author Contributions

T.W., J.L., H.Q., and W.S. contributed equally to this work. T.W., J.L., D.Z., P.C., and G.R. designed the study. T.W. wrote the manuscript, developed the figures, and conducted the analyses. H.Q. performed gene‐level analyses and constructed predictive models. J.L., W.S., and Y.L. did the analyses and developed figures. M.L., Z.L., and P.C. developed analytical skills and interpreted the results. M.L., and Z.L. extracted data from the website. H.W. reviewed the literature and provided materials. Y.Z., and L.Z. offered academic advice. All authors commented on the manuscript and approved the final version. D.Z. served as the guarantor for the overall content.

## Ethics Approval and Consent to Participate

All individuals provided written informed consent. North West Multi‐centre Research Ethics Committee approved the UK Biobank ethical application. Patients samples in this study were approved by the Committees for the Ethical Review of Research Involving Human Subjects at the Guangzhou Institute of Dermatology. All animal experiments conducted in this study were approved by the Welfare and Ethical Committee for Experimental Animal Care of Southern Medical University.

## Supporting information



Supporting Figure 1‐9

Supporting Table 1‐13

Supporting Table 14‐25

## Data Availability

Summary statistics from our MR analysis have been listed in Supplementary Table S1. The analysis code is available on GitHub (https://github.com/Nickxqq/PSO_LBGM). Individual‐level data from the UK Biobank requires approved access through their standard application process.
