# Supplementary material for: Population‐Based Multi‐Omics and Cohort Study Identifying Predictive Biomarkers and Therapeutic Targets for Psoriatic Disease
Source: Adv Sci (Weinh). 2025 Dec 2;13(8):e14130. doi: 10.1002/advs.202514130 (PMC12884755; doi:10.1002/advs.202514130)
Supplement: Supplementary file 1 — Supporting Figure 1‐9 [file ADVS-13-e14130-s002.pdf]

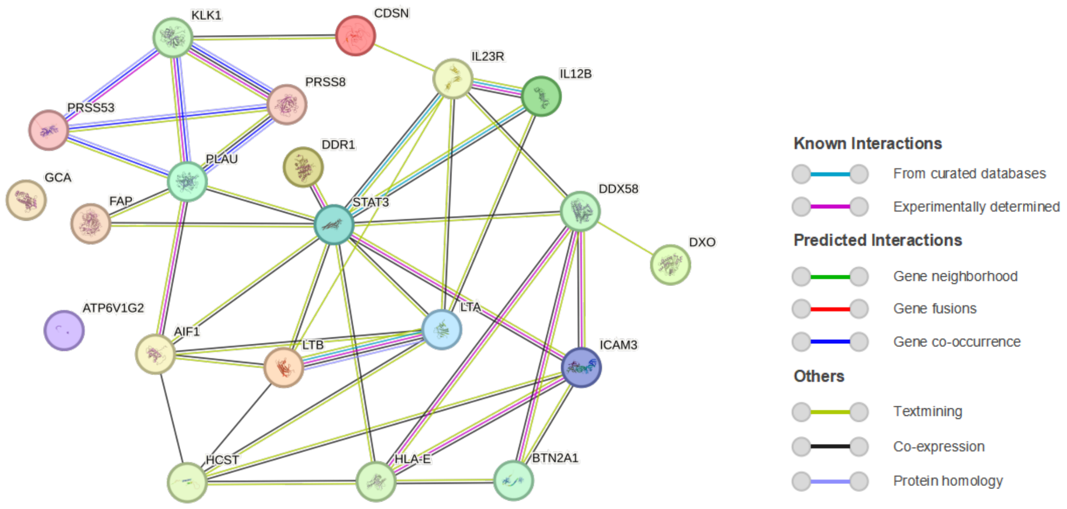

Supplementary Figure 1. Protein–protein interaction (PPI) networks of the MR-prioritized proteins. AIF1, allograft inflammatory factor 1; ATP6V1G2, V-type proton ATPase subunit G2; BTN2A1, butyrophilin subfamily 2 member A1; CDSN, corneodesmosin; DDR1, epithelial discoidin domain-containing receptor 1; DXO, decapping and exoribonuclease protein; FAP, prolyl endopeptidase; GCA, grancalcin; HCG22, protein PBMUCL2; HLA-E, HLA class I histocompatibility antigen, alpha chain E; ICAM3, intercellular adhesion molecule 3; IL12B, interleukin-12 subunit beta; IL23R, interleukin-23 receptor; KLK1, kallikrein-1; LTA, lymphotoxin-alpha; LTB, lymphotoxin-beta; MICA/MICB, MHC class I polypeptide-related sequence A/B; PLAU, urokinase-type plasminogen activator; PRSS53, serine protease 53; PRSS8, prostasin; RIGI, antiviral innate immune response receptor RIG-I; STAT3, signal transducer and activator of transcription 3.

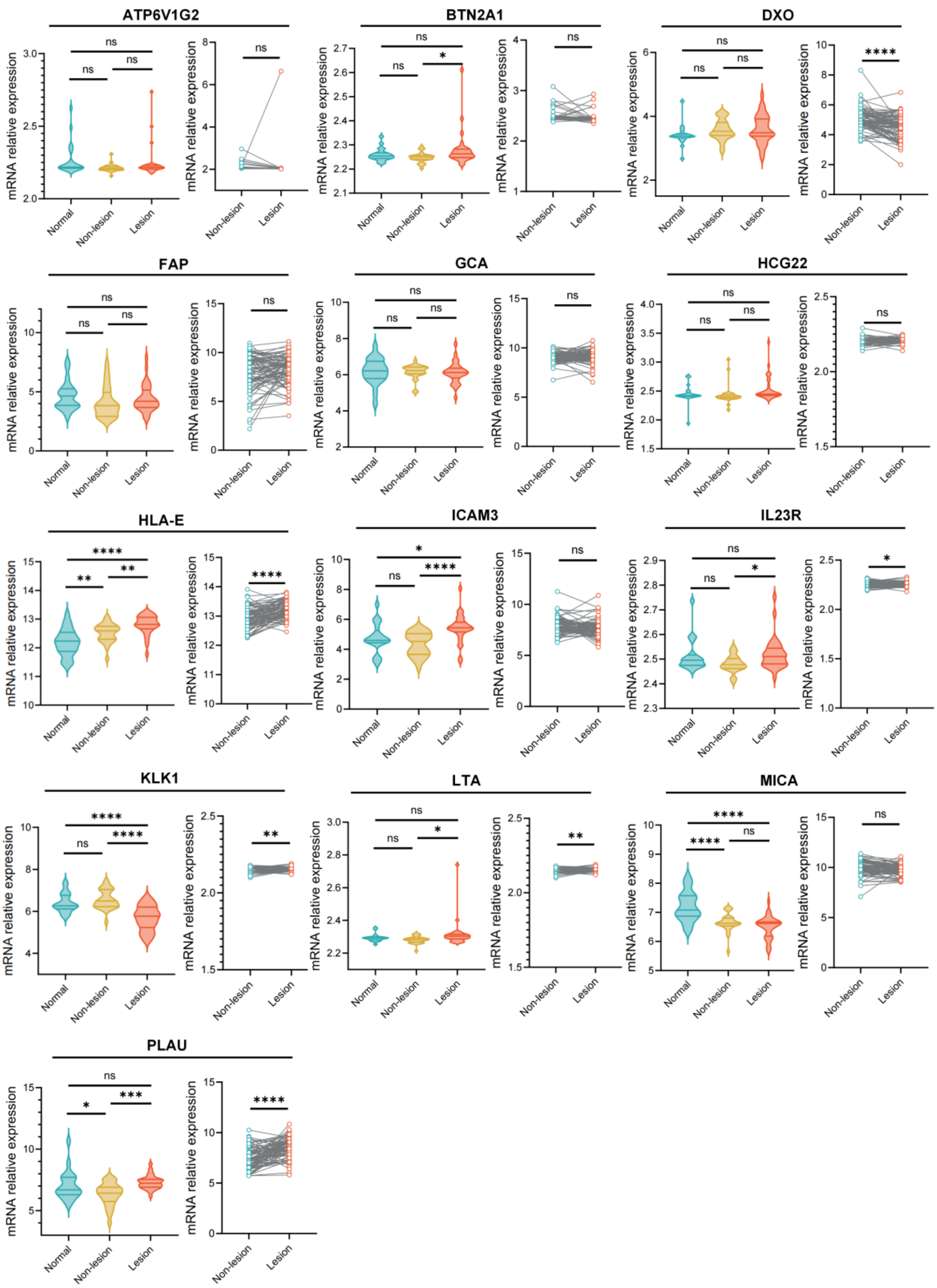
ATP6V1G2
BTN2A1
DXO
FAP
GCA
HCG22
HLA-E
ICAM3
IL23R
KLK1
LTA
MICA
PLAU
mRNA relative expression
Normal
Non-lesion
Lesion
ns
*
**
***
****

Supplementary Figure 2. Expression of mRNA in skin tissue from normal donors, lesional and non-lesional PsD skin. The GSE14905 dataset comprised skin samples from 21 normal donors, lesional skin from 29 PsD patients, and non-lesional skin from 26 PsD patients. Group comparisons were performed using one-way ANOVA. The GSE30999 dataset included lesional and non-lesional skin samples from 85 paired PsD patients, with paired t-tests employed for inter-group comparisons. ATP6V1G2, V-type proton ATPase subunit G2; BTN2A1, butyrophilin subfamily 2 member A1; DXO, decapping and exoribonuclease protein; FAP, prolyl endopeptidase; GCA, grancalcin; HCG22, protein PBMUCL2; HLA-E, HLA class I histocompatibility antigen, alpha chain E; ICAM3, intercellular adhesion molecule 3; IL23R, interleukin-23 receptor; KLK1, kallikrein-1; LTA, lymphotoxin-alpha; MICA, MHC class I polypeptide-related sequence A; PLAU, urokinase-type plasminogen activator.

A AIF1

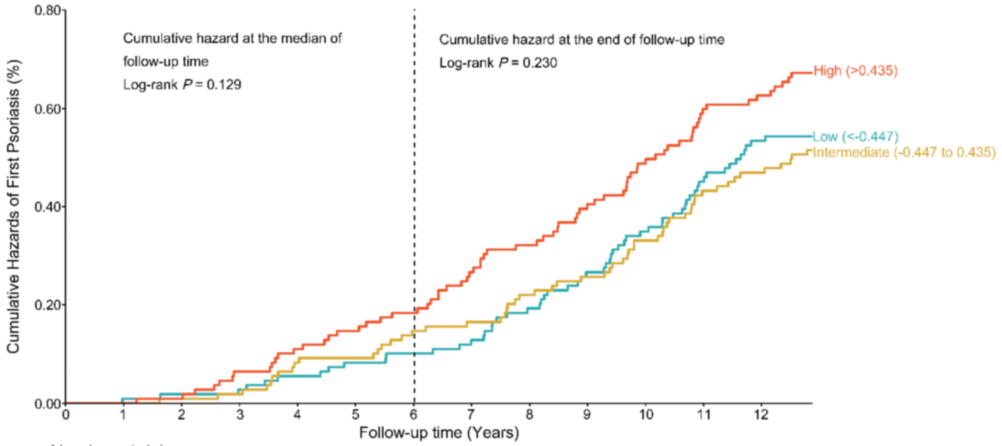

Number at risk

10893	10892	10891	10890	10887	10884	10882	10879	10872	10864	10855	10844	10835
10895	10895	10894	10893	10886	10885	10879	10877	10871	10867	10859	10848	10844
10892	10892	10891	10885	10880	10876	10872	10863	10857	10848	10839	10827	10824

B ATP6V1G2

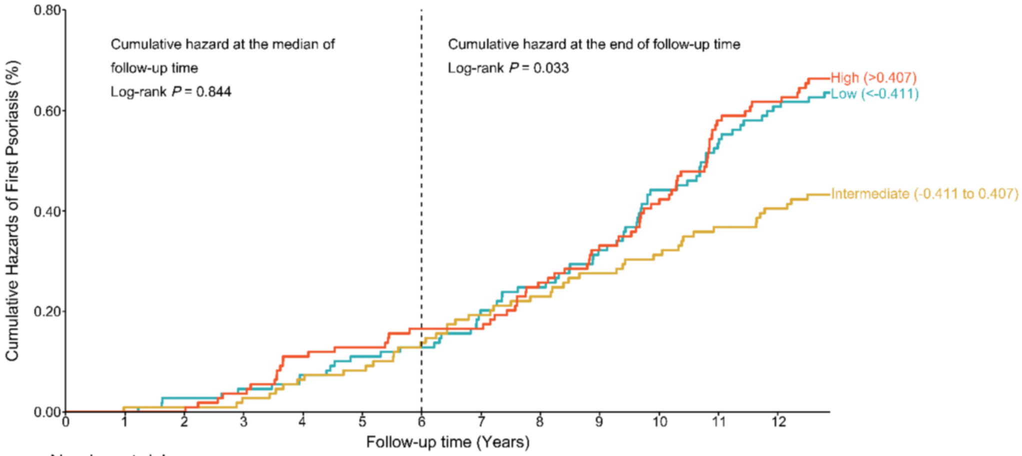

Number at risk

10893	10893	10890	10888	10885	10881	10879	10871	10866	10858	10845	10835	10827
10895	10894	10894	10892	10888	10886	10880	10874	10870	10865	10861	10855	10851
10892	10892	10892	10888	10880	10878	10874	10874	10864	10856	10847	10829	10825

C BTN2A1

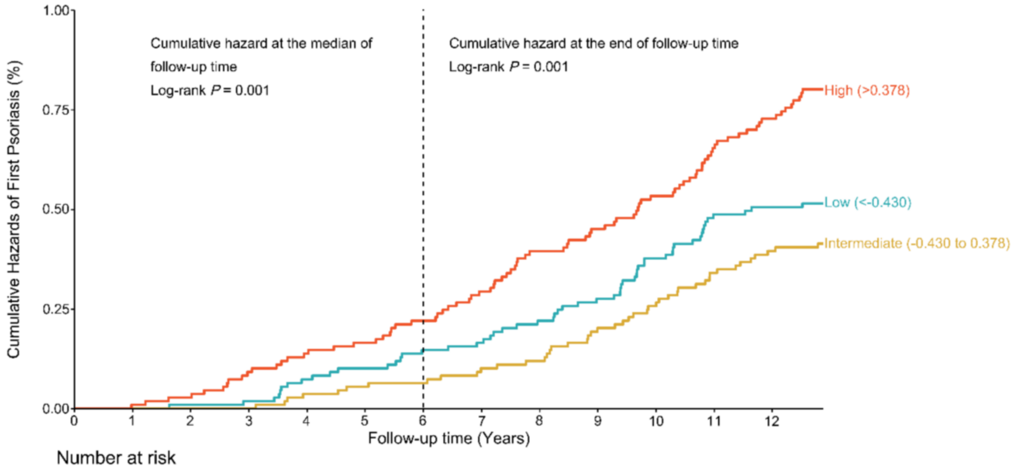

Number at risk

10898	10898	10897	10896	10890	10887	10882	10880	10874	10868	10857	10845	10843
10888	10888	10888	10888	10884	10882	10881	10877	10875	10866	10860	10851	10845
10894	10893	10891	10884	10879	10876	10870	10862	10851	10845	10836	10823	10815

D DDR1

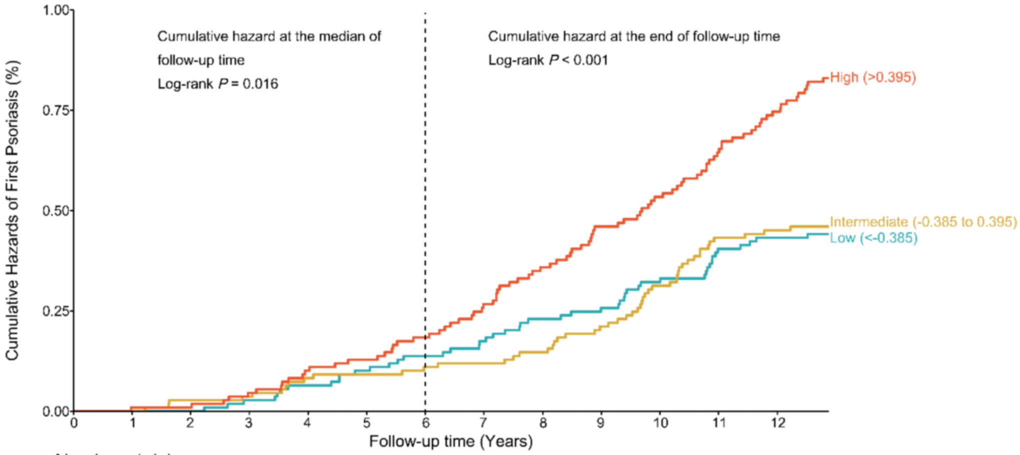

Number at risk

10895	10895	10895	10892	10888	10884	10880	10876	10870	10867	10860	10851	10848
10893	10893	10890	10889	10884	10883	10881	10880	10877	10870	10859	10846	10844
10892	10891	10891	10887	10881	10878	10872	10863	10853	10842	10834	10822	10811

E DXO

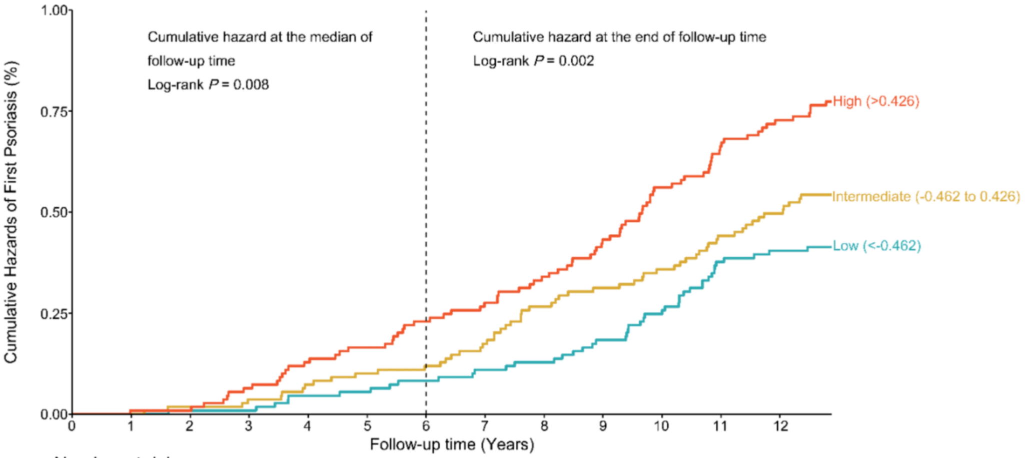

Number at risk

10893	10893	10892	10892	10888	10887	10884	10881	10879	10873	10866	10852	10849
10892	10892	10890	10888	10884	10881	10879	10873	10863	10858	10853	10844	10838
10895	10894	10894	10888	10881	10877	10870	10865	10858	10848	10834	10823	10816

F FAP

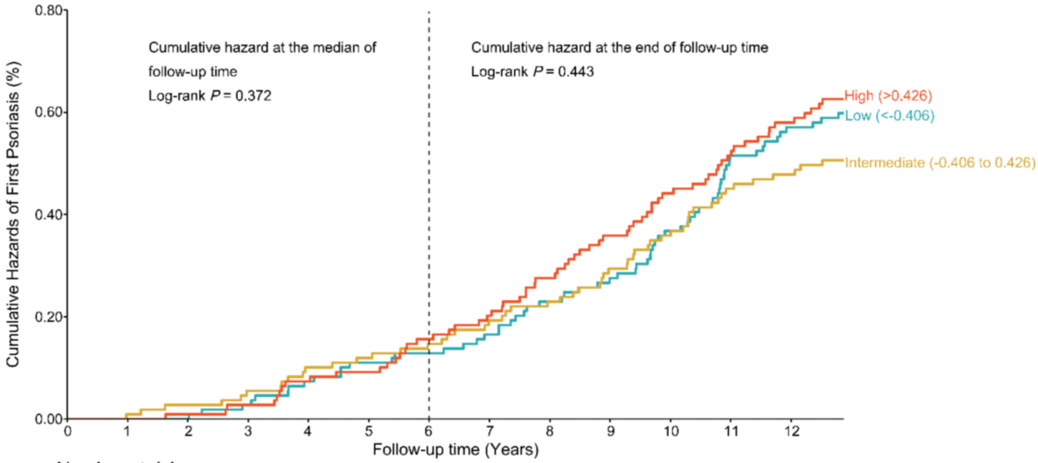

Number at risk

10894	10894	10894	10891	10886	10882	10880	10876	10869	10864	10854	10838	10832
10893	10892	10890	10887	10882	10880	10877	10872	10868	10861	10854	10844	10841
10893	10893	10892	10890	10885	10883	10876	10871	10863	10854	10845	10837	10830

G HCG22

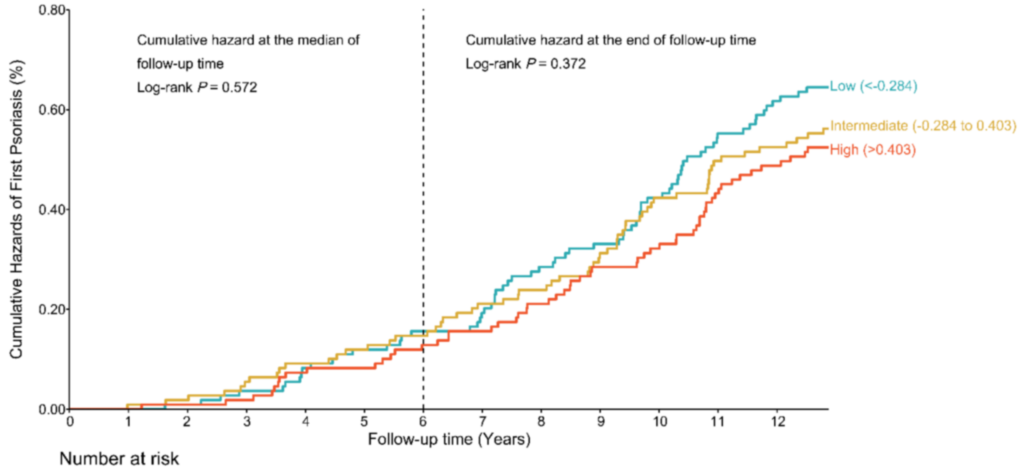

Number at risk

10893	10893	10892	10889	10884	10880	10876	10872	10862	10857	10847	10833	10826
10892	10891	10890	10886	10882	10879	10876	10869	10866	10858	10846	10838	10835
10895	10895	10894	10893	10887	10886	10881	10878	10872	10864	10860	10848	10842

H ICAM3

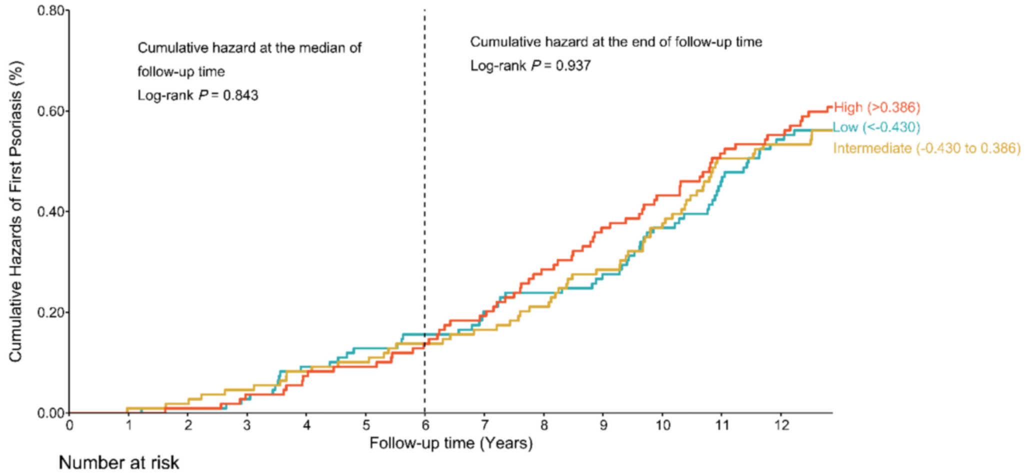

Number at risk

10893	10893	10892	10890	10883	10879	10876	10871	10867	10863	10853	10843	10834
10898	10897	10896	10893	10889	10887	10883	10880	10875	10867	10858	10843	10840
10889	10889	10888	10885	10881	10879	10874	10868	10858	10849	10842	10833	10829

I IL12B

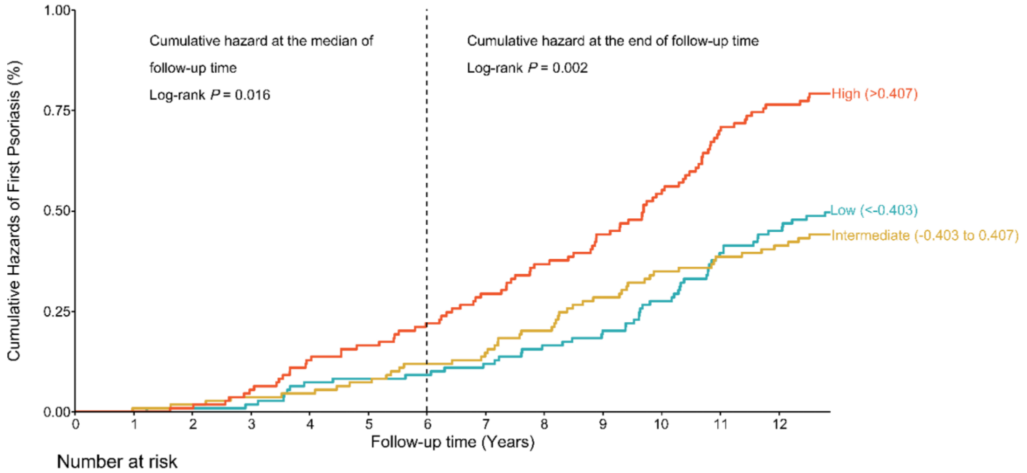

Number at risk

10893	10893	10892	10891	10885	10884	10883	10880	10875	10871
10892	10891	10890	10888	10887	10884	10879	10876	10870	10861
10895	10895	10894	10889	10881	10877	10871	10863	10855	10847

J KLK1

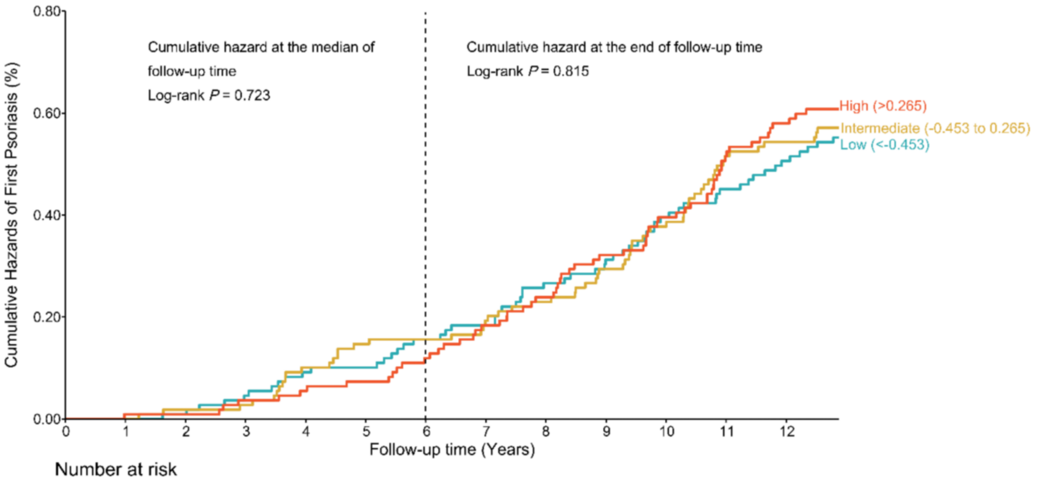

Number at risk

10896	10896	10895	10891	10886	10885	10879	10876	10867	10862	10853	10847	10841
10890	10890	10888	10887	10879	10874	10873	10869	10865	10858	10849	10834	10831
10894	10893	10893	10890	10888	10886	10881	10874	10868	10859	10851	10838	10831

K LTA

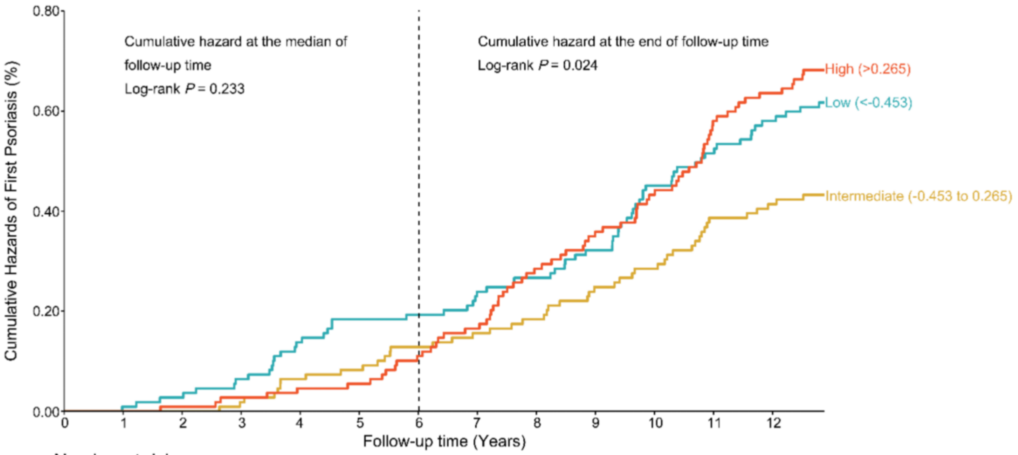

Number at risk

10893	10892	10890	10886	10878	10873	10872	10867	10864	10858	10844	10837	10830
10894	10894	10894	10892	10887	10885	10880	10877	10874	10867	10863	10852	10849
10893	10893	10892	10890	10888	10887	10881	10875	10862	10854	10846	10830	10824

L LTB

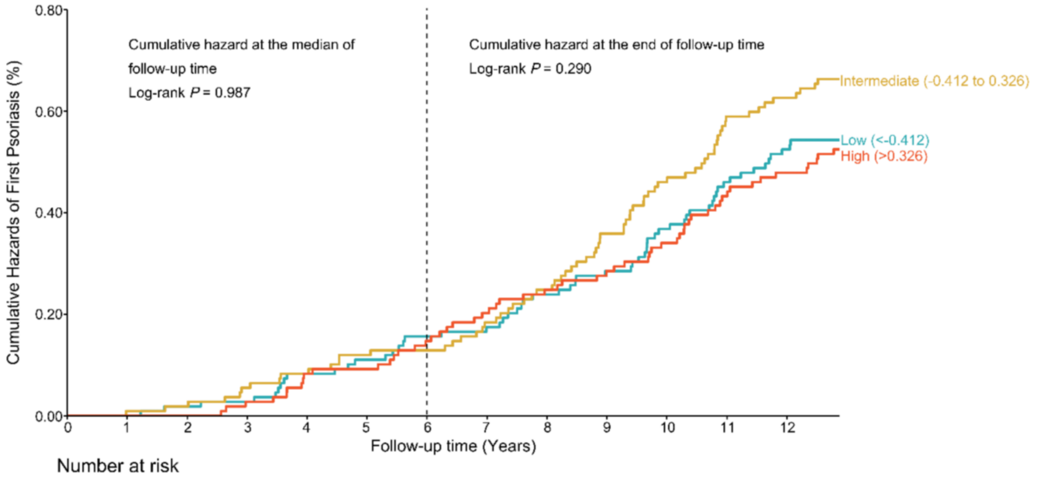

Number at risk

10896	10896	10894	10893	10887	10884	10879	10877	10870	10865	10856	10846	10839
10894	10893	10892	10888	10885	10881	10880	10874	10867	10855	10844	10830	10826
10890	10890	10890	10887	10881	10880	10874	10868	10863	10859	10853	10843	10838

M MICA/MICB

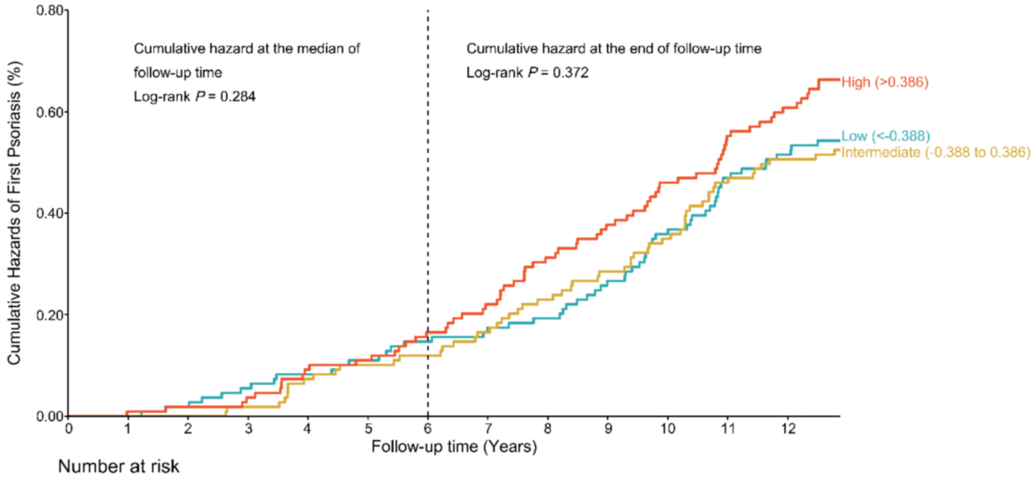

Number at risk

10894	10894	10892	10888	10885	10882	10878	10875	10873	10865	10855	10843	10838
10891	10891	10891	10889	10883	10880	10878	10873	10866	10860	10853	10841	10836
10895	10894	10893	10891	10885	10883	10877	10871	10861	10854	10845	10835	10829

N PLAU

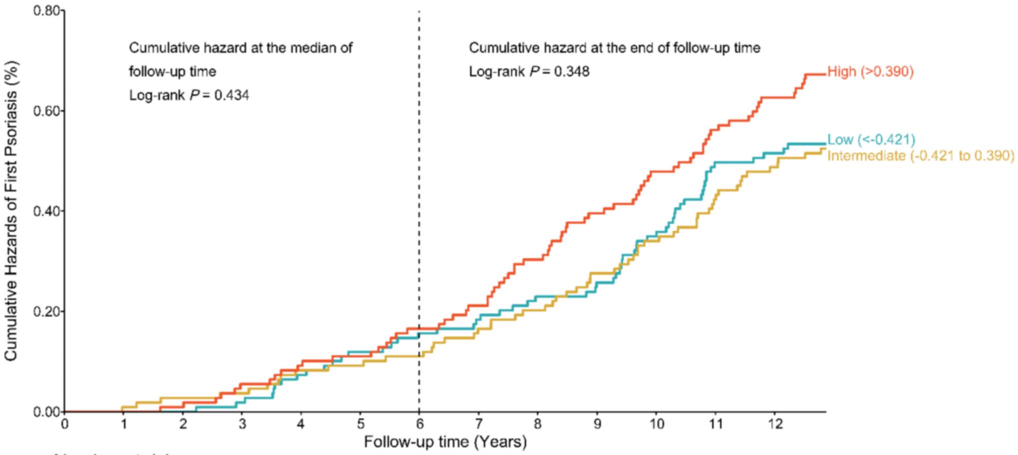

Number at risk

10893	10893	10893	10891	10885	10880	10876	10873	10868	10865	10855	10839	10837
10895	10894	10892	10891	10886	10885	10883	10877	10873	10865	10858	10849	10842
10892	10892	10891	10886	10882	10880	10874	10869	10859	10849	10840	10831	10824

L PRSS53

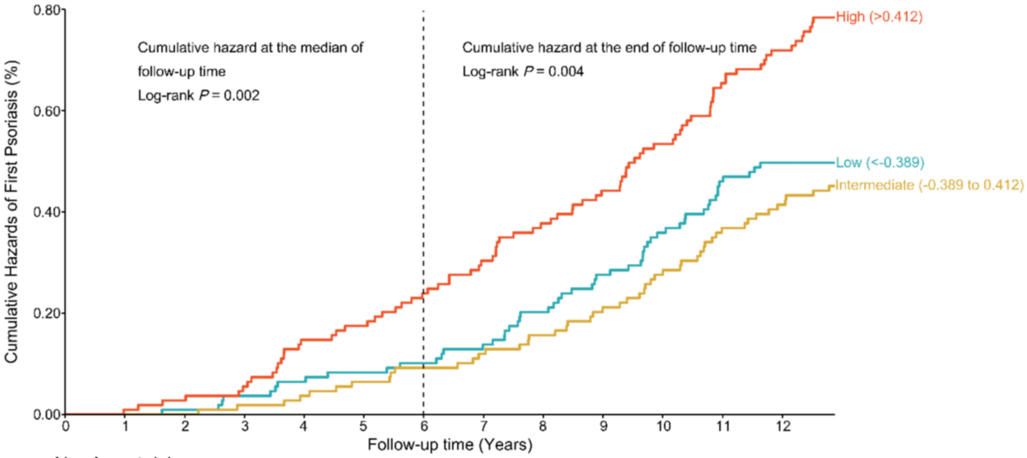

Number at risk

10897	10897	10896	10893	10890	10888	10886	10882	10875	10867	10858	10847	10843
10891	10891	10891	10889	10887	10884	10881	10878	10874	10868	10861	10851	10846
10892	10891	10889	10886	10876	10873	10866	10859	10851	10844	10834	10821	10814

Supplementary Figure 3. Kaplan-Meier (KM) curves for PsD event timing across three strata of candidate protein levels. The expression levels of candidate proteins were categorized into tertiles, representing high, medium, and low expression groups. KM curves were employed to illustrate the risk stratification capability of the protein biomarkers, with group comparisons conducted using the Log-rank test. Raw, unadjusted protein levels were used. AIF1, allograft inflammatory factor 1; ATP6V1G2, V-type proton ATPase subunit G 2; BTN2A1, butyrophilin subfamily 2 member A1; DDR1, epithelial discoidin domain-containing receptor 1; DXO, decapping and exoribonuclease protein; FAP, prolyl endopeptidase; HCG22, protein PBMUCL2; ICAM3, intercellular adhesion molecule 3; IL12B, interleukin-12 subunit beta; KLK1, kallikrein-1; LTA, lymphotoxin-alpha; LTB, lymphotoxin-beta; MICA/MICB, MHC class I polypeptide-related sequence A/B; PLAU, urokinase-type plasminogen activator; PRSS53, serine protease 53.

A

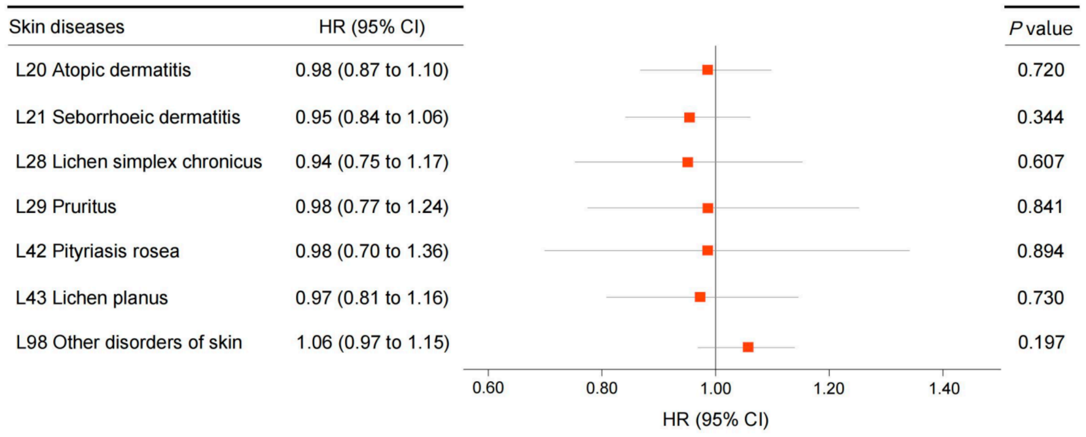

B

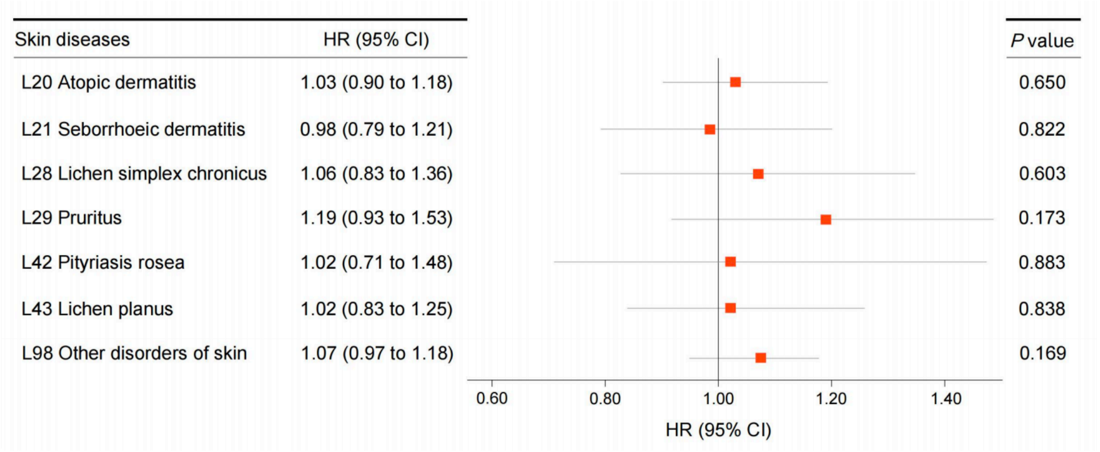

Supplementary Figure 4. Asssociations between CDSN (J), PRSS8 (K) and multiple diseases requiring differential diagnosis with PsD. The analysis was conducted using multivariable Cox proportional hazards regression, adjusting for age, sex, body mass index (BMI), and batch of protein assay.

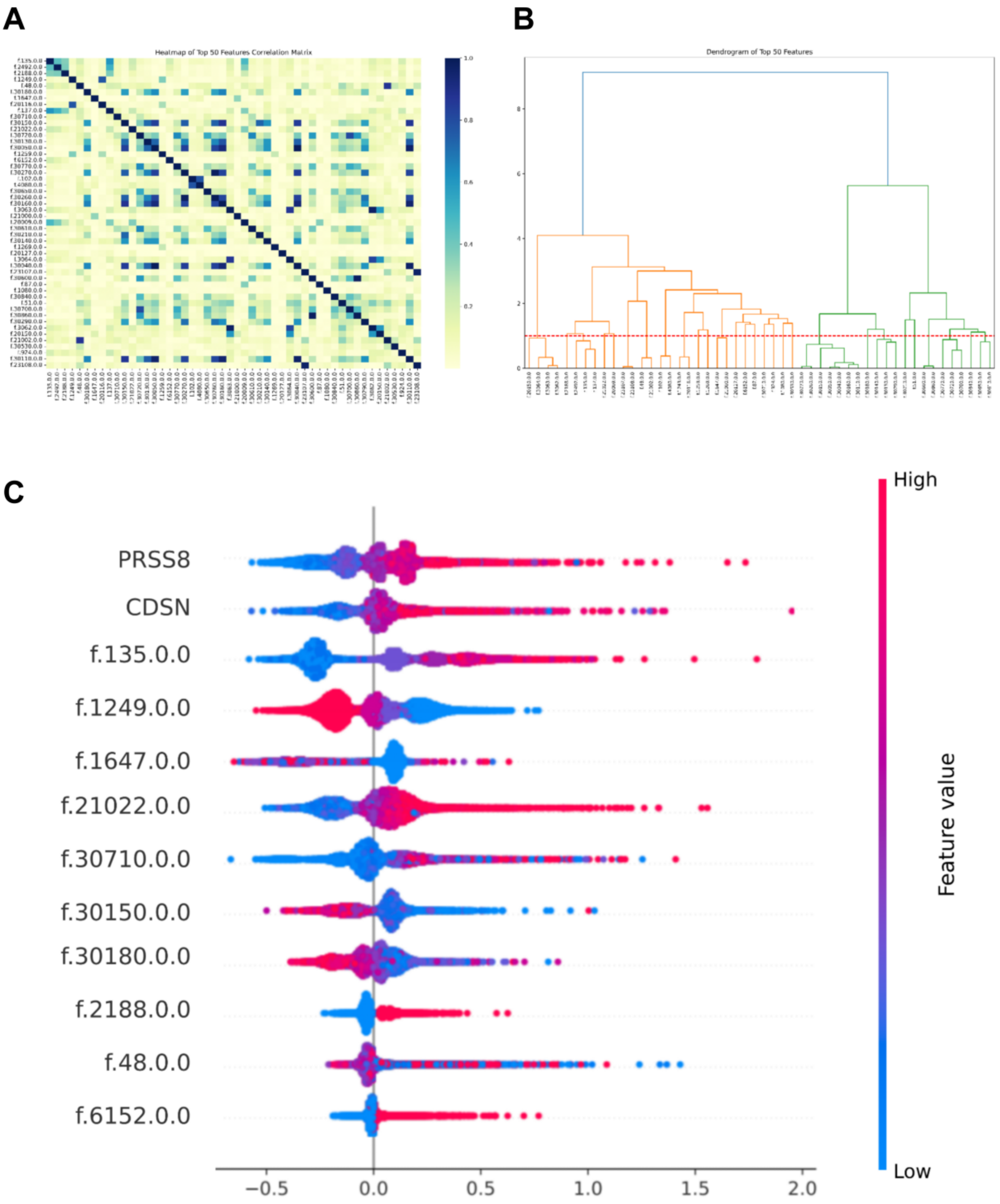

Supplementary Figure 5. The correlation heatmap (A), clustering diagram (B), and SHAP plot (C) for clinical predictor factor selection. Hierarchical clustering was conducted using Spearman rank-order correlations to mitigate multicollinearity. An arbitrary threshold of 1.00 was then applied to prune the dendrogram, retaining only one predictor from each cluster where predictors fell below this threshold. SHapley Additive exPlanations (SHAP) plot was used to visualize the contribution of each predictor to incident PsD. The corresponding objects for Field ID numbers can be found on the UK Biobank official website.

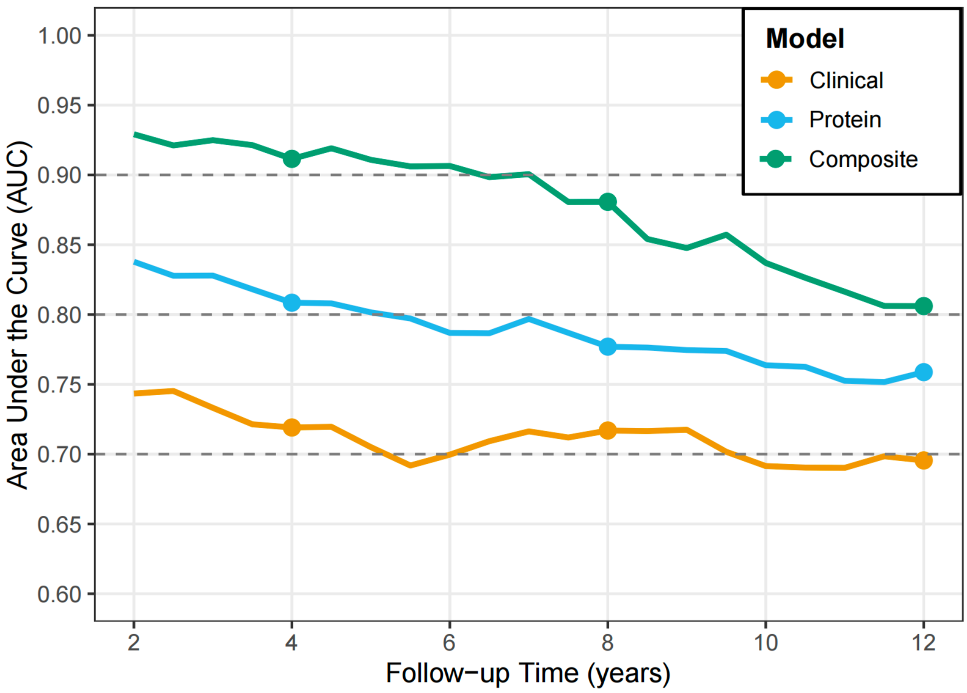

Supplementary Figure 6. Time-dependent area under the curve (AUC) values for clinical, protein, and composite prediction models across 2-12 years of follow-up in the UK Biobank cohort.

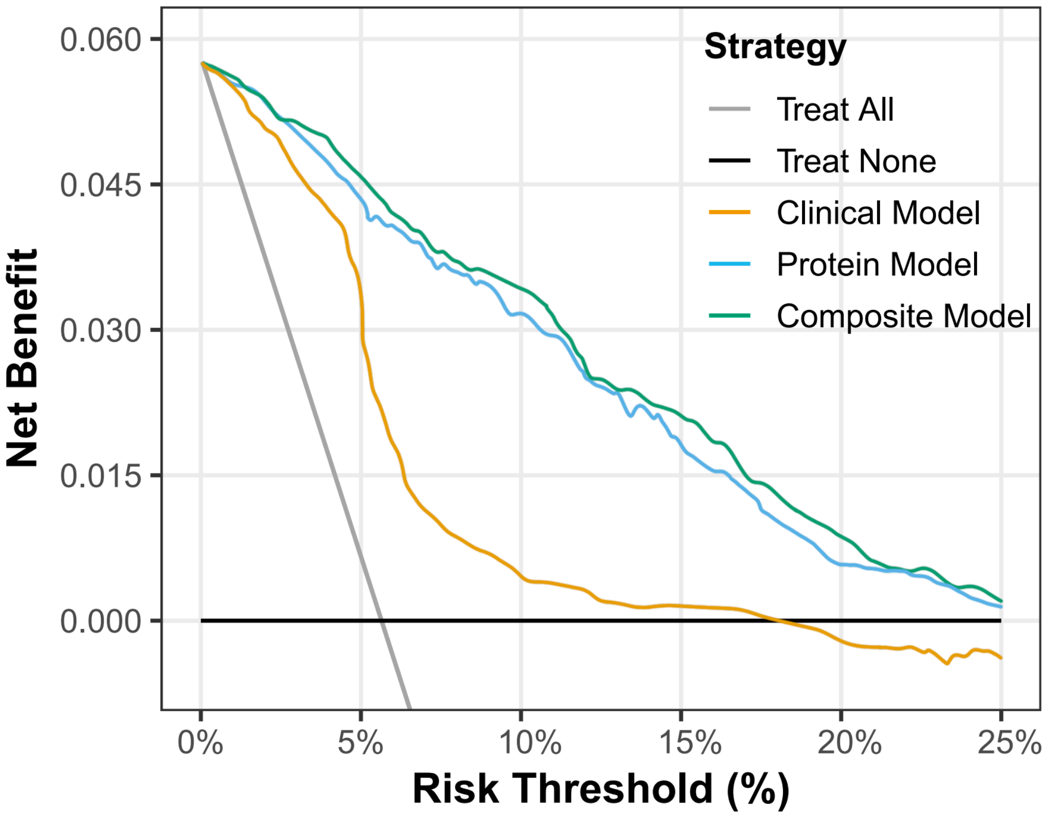

Supplementary Figure 7. Decision curve analysis for psoriatic arthritis (PsD) risk prediction models. The y-axis represents the net benefit. The x-axis represents the threshold probability, indicating the minimum risk at which a clinician would recommend confirmatory evaluation for PsD. The gray line represents the net benefit of evaluating all patients ("treat all"), while the black line (y=0) represents evaluating no patients ("treat none"). Colored lines represent different prediction strategies: Clinical Model (orange), Protein Model (blue), and Composite Model (green).

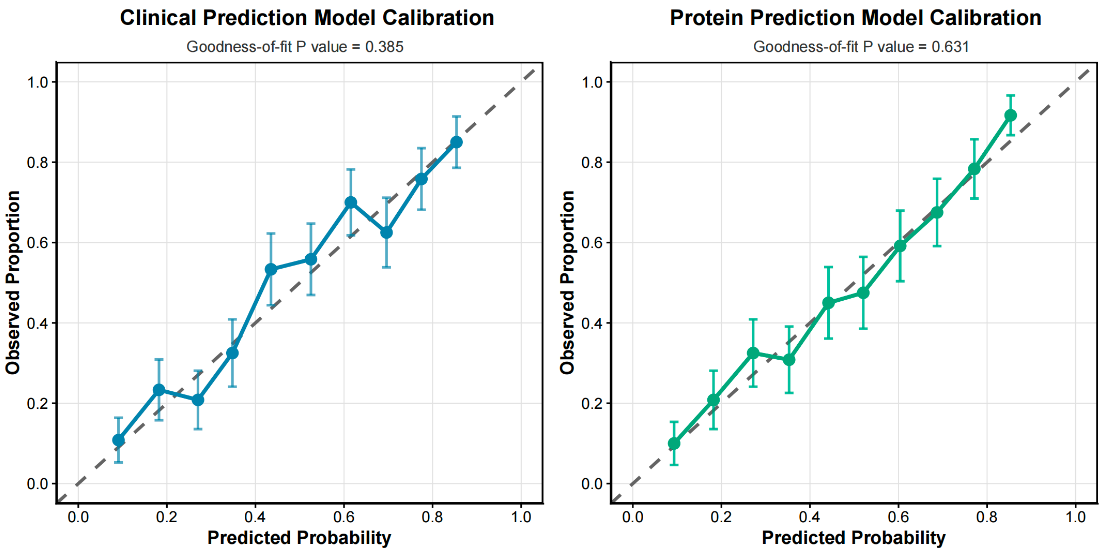

Supplementary Figure 8. Calibration curves for clinical and protein-based prediction models. This figure displays calibration plots assessing the agreement between predicted probabilities and observed proportions of PsD for two prediction models. The left panel presents calibration performance for the clinical prediction model, which incorporates traditional clinical risk factors and demographic variables. The right panel illustrates calibration performance for the protein prediction model.

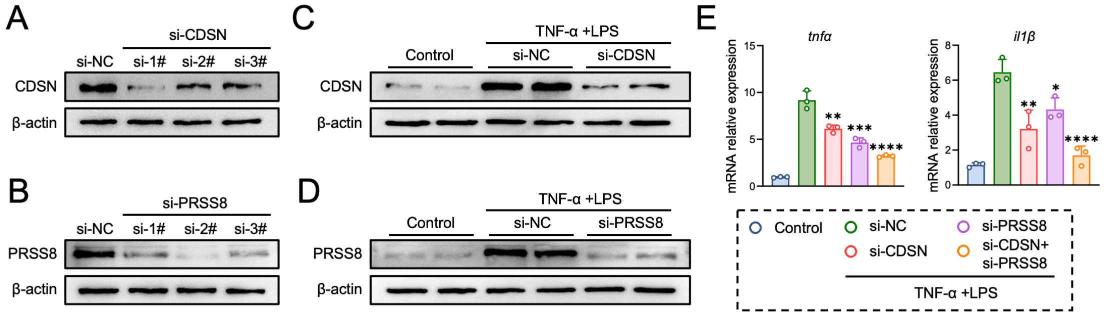

Supplementary Figure 9. (A-B) The validation of protein levels of CDSN (A) and PRSS8 (B) in HaCaT after si-RNA knockdown. (C-D) Stimulated by TNF-α plus LPS, the protein levels of CDSN (C) and PRSS8 (D) in HaCaT after si-RNA knockdown. (E) Relative mRNA expression levels of the pro-inflammatory cytokines in HaCaT.
