# Supplementary material for: Population‐Based Multi‐Omics and Cohort Study Identifying Predictive Biomarkers and Therapeutic Targets for Psoriatic Disease
Source: Adv Sci (Weinh). 2025 Dec 2;13(8):e14130. doi: 10.1002/advs.202514130 (PMC12884755; doi:10.1002/advs.202514130)
Supplement: Supplementary file 3 — Supporting Table 14‐25 [file ADVS-13-e14130-s001.pdf]

Supplementary Table 14. Characteristics of participants with and without PsD in the UKB-PPP

**Characteristic**	**PsD (n=192)**	**Non-PsD (n=32,488)**	***P*-value**
Age (years)	59.94 ± 7.06	56.96 ± 8.20	**<0.001**
BMI (kg/m^2)	28.65 ± 5.35	27.47 ± 4.79	**0.001**
Sex			**0.026**
Female	90 (46.88)	17,833 (54.89)	
Male	102 (53.12)	14,655 (45.11)	
Ethnicity (White)	176 (91.7)	29,242 (90.0)	0.672
AIF1	1.35 ± 1.02	1.28 ± 1.05	0.317
ATP6V1G2	1.37 ± 1.40	1.30 ± 1.31	0.446
BTN2A1	1.45 ± 1.07	1.30 ± 1.42	0.174
CDSN	1.53 ± 1.25	1.28 ± 1.17	**<0.001**
DDR1	1.58 ± 1.27	1.31 ± 2.80	0.172
DXO	1.62 ± 1.36	1.27 ± 0.97	**<0.001**
FAP	1.26 ± 0.90	1.27 ± 1.02	0.868
HCG22	1.25 ± 1.14	1.29 ± 1.39	0.695
ICAM3	1.38 ± 1.37	1.30 ± 1.59	0.481
IL12B	1.44 ± 1.19	1.28 ± 1.09	**0.034**
KLK1	1.31 ± 0.92	1.26 ± 0.88	0.488
LTA	1.44 ± 2.98	1.28 ± 1.49	0.144
LTB	1.21 ± 1.38	1.49 ± 4.88	0.435
MICA / MICB	1.50 ± 1.44	1.29 ± 1.44	**0.041**
PLAU	1.39 ± 1.07	1.36 ± 4.45	0.932
PRSS53	1.40 ± 1.05	1.32 ± 2.65	0.684
PRSS8	1.73 ± 1.26	1.27 ± 1.02	**<0.001**

Data were presented as numbers (percentages) or mean value ± standard deviation (SD). The unit for protein expression level is normalized protein expression (NPX). The NPX values are measured on the $\log_2$ scale, with lower values indicating lower levels of the analyte. Categorical variables were analyzed using a two-sided χ^2 test. Continuous data were analyzed using a two-sided t-test. *P*-value < 0.05 was considered as significant. UKB-PPP, UK Biobank Pharma Proteomics Project; PsD, psoriatic disease; BMI, body mass index; AIF1, allograft inflammatory factor 1; ATP6V1G2, V-type proton ATPase subunit G 2; BTN2A1, butyrophilin subfamily 2 member A1; CDSN, corneodesmosin; DDR1, epithelial discoidin domain-containing receptor 1; DXO, decapping and exoribonuclease protein; FAP, prolyl endopeptidase; HCG22, protein PBMUCL2; ICAM3, intercellular adhesion molecule 3; IL12B, interleukin-12 subunit beta; KLK1, kallikrein-1; LTA, lymphotoxin-alpha; LTB, lymphotoxin-beta; MICA/MICB, MHC class I polypeptide-related sequence A/B; PLAU, urokinase-type plasminogen activator; PRSS53, serine protease 53; PRSS8, prostasin.

Supplementary Table 15. Association between candidate proteins and incident PsD risks in the UKB-PPP

	≤ 2 years		≤ 4 years		≤ 6 years		≤ 8 years		≤ 10 years		≤ 12 years	
**Proteins**	**HR (95% CI)**	***P*-value**	**HR (95% CI)**	***P*-value**	**HR (95% CI)**	***P*-value**	**HR (95% CI)**	***P*-value**	**HR (95% CI)**	***P*-value**	**HR (95% CI)**	***P*-value**
AIF1	1.16 (0.79, 1.70)	0.441	1.29 (0.96, 1.70)	0.081	1.15 (0.91, 1.46)	0.245	1.05 (0.89, 1.24)	0.568	1.02 (0.88, 1.19)	0.767	1.02 (0.88, 1.18)	0.812
ATP6V1G2	1.04 (0.70, 1.54)	0.857	0.96 (0.72, 1.28)	0.763	1.01 (0.79, 1.29)	0.936	1.01 (0.85, 1.20)	0.910	0.96 (0.82, 1.13)	0.641	0.95 (0.81, 1.11)	0.504
BTN2A1	1.17 (0.78, 1.75)	0.456	1.28 (0.95, 1.73)	0.103	1.25 (0.97, 1.61)	0.087	1.02 (0.86, 1.21)	0.836	1.05 (0.89, 1.24)	0.545	1.05 (0.89, 1.23)	0.557
DDR1	1.06 (0.71, 1.59)	0.770	1.25 (0.93, 1.69)	0.139	1.29 (1.00, 1.66)	0.052	1.21 (1.01, 1.44)	**0.037**	1.29 (1.09, 1.52)	**0.003**	1.26 (1.07, 1.48)	**0.005**
DXO	1.48 (1.00, 2.19)	**0.050**	1.36 (1.03, 1.81)	**0.033**	1.35 (1.06, 1.72)	**0.014**	1.22 (1.04, 1.44)	**0.017**	1.23 (1.05, 1.43)	**0.009**	1.22 (1.05, 1.42)	**0.009**
FAP	0.94 (0.64, 1.39)	0.763	1.08 (0.81, 1.43)	0.609	1.15 (0.90, 1.46)	0.273	0.97 (0.82, 1.15)	0.715	0.98 (0.84, 1.15)	0.823	1.00 (0.85, 1.16)	0.955
HCG22	0.81 (0.53, 1.22)	0.313	0.91 (0.67, 1.24)	0.558	0.86 (0.66, 1.12)	0.257	0.94 (0.78, 1.12)	0.485	0.95 (0.80, 1.12)	0.538	0.93 (0.79, 1.09)	0.371
ICAM3	1.06 (0.72, 1.57)	0.758	1.17 (0.88, 1.56)	0.274	1.17 (0.92, 1.49)	0.213	1.05 (0.89, 1.25)	0.544	1.05 (0.90, 1.23)	0.507	1.04 (0.89, 1.21)	0.650
IL12B	1.11 (0.74, 1.65)	0.619	1.36 (1.01, 1.83)	**0.043**	1.25 (0.97, 1.61)	0.083	1.21 (1.02, 1.44)	**0.031**	1.15 (0.98, 1.35)	0.081	1.16 (0.99, 1.36)	0.064
KLK1	0.85 (0.59, 1.24)	0.405	0.93 (0.71, 1.22)	0.597	0.90 (0.72, 1.13)	0.368	1.01 (0.87, 1.18)	0.878	1.02 (0.88, 1.18)	0.810	1.03 (0.89, 1.18)	0.729
LTA	0.65 (0.43, 0.98)	**0.040**	0.88 (0.66, 1.19)	0.407	0.97 (0.76, 1.25)	0.827	1.05 (0.88, 1.25)	0.616	1.06 (0.90, 1.24)	0.495	1.06 (0.91, 1.25)	0.448
LTB	0.92 (0.61, 1.40)	0.698	0.95 (0.70, 1.29)	0.740	0.90 (0.69, 1.17)	0.423	0.88 (0.74, 1.06)	0.171	0.89 (0.75, 1.05)	0.176	0.87 (0.74, 1.03)	0.111
MICA/MICB	0.82 (0.55, 1.22)	0.327	1.10 (0.82, 1.47)	0.532	1.20 (0.94, 1.55)	0.150	1.06 (0.89, 1.26)	0.523	1.04 (0.89, 1.22)	0.607	1.05 (0.90, 1.23)	0.550
PLAU	1.05 (0.70, 1.57)	0.809	1.30 (0.97, 1.75)	0.078	1.35 (1.05, 1.74)	**0.020**	1.10 (0.92, 1.31)	0.294	1.17 (1.00, 1.37)	0.057	1.14 (0.97, 1.34)	0.106
PRSS53	1.25 (0.84, 1.86)	0.266	1.38 (1.03, 1.85)	**0.032**	1.32 (1.03, 1.69)	**0.030**	1.18 (1.00, 1.41)	0.053	1.22 (1.04, 1.42)	**0.016**	1.19 (1.02, 1.40)	**0.028**

Note that the PsD cases in each group were cumulative. Hazard ratio (HR) indicates the change in the risk of developing PsD associated with a 1-standard deviation (SD) increase in protein expression, adjusted for age, sex, body mass index (BMI), and batch of protein assay. PsD, psoriatic disease; UKB-PPP, UK Biobank Pharma Proteomics Project; AIF1, allograft inflammatory factor 1; ATP6V1G2, V-type proton ATPase subunit G 2; BTN2A1, butyrophilin subfamily 2 member A1; DDR1, epithelial discoidin domain-containing receptor 1; DXO, decapping and exoribonuclease protein; FAP, prolyl endopeptidase; HCG22, protein PBMUCL2; ICAM3, intercellular adhesion molecule 3; IL12B, interleukin-12 subunit beta; KLK1, kallikrein-1; LTA, lymphotoxin-alpha; LTB, lymphotoxin-beta; MICA/MICB, MHC class I polypeptide-related sequence A/B; PLAU, urokinase-type plasminogen activator; PRSS53, serine protease 53.

Supplementary Table 16. Discriminative capacity of candidate proteins for PsD events in the UKB-PPP

**Proteins**	**AUC (95% CI)**	***P*-value**
AIF1	0.51 (0.39, 0.64)	0.856
ATP6V1G2	0.54 (0.42, 0.66)	0.489
BTN2A1	0.60 (0.47, 0.73)	0.082
**CDSN**	**0.64 (0.53, 0.75)**	**0.017**
DDR1	0.52 (0.39, 0.64)	0.782
DXO	0.61 (0.48, 0.73)	0.063
FAP	0.50 (0.38, 0.61)	0.959
HCG22	0.51 (0.41, 0.60)	0.922
ICAM3	0.47 (0.35, 0.59)	0.621
IL12B	0.57 (0.45, 0.70)	0.204
KLK1	0.43 (0.32, 0.55)	0.246
**LTA**	**0.65 (0.54, 0.76)**	**0.008**
LTB	0.49 (0.38, 0.59)	0.797
MICA/MICB	0.46 (0.34, 0.59)	0.522
PLAU	0.52 (0.39, 0.64)	0.778
**PRSS8**	**0.66 (0.55, 0.77)**	**0.006**
PRSS53	0.58 (0.46, 0.70)	0.145

All tests were two-sided. AUC >0.50 and $P < 0.05$ were considered as significant.PsD, psoriatic disease; UKB-PPP, UK Biobank Pharma Proteomics Project; AUC, area under the receiver operating characteristic curve; AIF1, allograft inflammatory factor 1; ATP6V1G2, V-type proton ATPase subunit G 2; BTN2A1, butyrophilin subfamily 2 member A1; CDSN, corneodesmosin; DDR1, epithelial discoidin domain-containing receptor 1; DXO, decapping and exoribonuclease protein; FAP, prolyl endopeptidase; HCG22, protein PBMUCL2; ICAM3, intercellular adhesion molecule 3; IL12B, interleukin-12 subunit beta; KLK1, kallikrein-1; LTA, lymphotoxin-alpha; LTB, lymphotoxin-beta; MICA/MICB, MHC class I polypeptide-related sequence A/B; PLAU, urokinase-type plasminogen activator; PRSS53, serine protease 53; PRSS8, prostasin.

Supplementary Table 17. Summary of analyses for identifying therapeutic targets and predictive biomarkers throughout the workflow

Proteins	Genetic prediction[1]	Transcriptional validation[2]	Population evaluation		
			**Baseline difference[3]**	**Risk stratification[4]**	**Discriminative capacity[5]**
AIF1	Not support	Support	Not support	Not support	Not support
ATP6V1G2	Not support	Not support	Not support	Support	Not support
BTN2A1	Not support	Not support	Not support	Support	Not support
**CDSN**	**Support**	**Support**	**Support**	**Support**	**Support**
DDR1	Not support	Support	Not support	Support	Not support
DXO	Not support	Not support	Support	Support	Not support
FAP	Support	Not support	Not support	Not support	Not support
GCA*	Not support	Not support	Not available	Not available	Not available
HCG22	Not support	Not support	Not support	Not support	Not support
HLA-E*	Not support	Support	Not available	Not available	Not available
ICAM3	Not support	Not support	Not support	Not support	Not support
IL12B	Not support	Support	Support	Support	Not support
IL23R*	Support	Not support	Not available	Not available	Not available
KLK1	Not support	Support	Not support	Not support	Not support
LTA	Not support	Not support	Not support	Support	Support
LTB	Not support	Support	Not support	Not support	Not support
MICA/MICB	Not support	Support	Support	Not support	Not support
PLAU	Not support	Not support	Not support	Not support	Not support
PRSS53	Support	Support	Not support	Support	Not support
**PRSS8**	**Support**	**Support**	**Support**	**Support**	**Support**
RIGI*	Support	Not support	Not available	Not available	Not available
STAT3*	Support	Support	Not available	Not available	Not available

[1]In the 'genetic prediction' section, the supporting criteria are that the candidate protein passes Mendelian randomization analysis.

[2]In the 'transcriptional validation' section, the supporting criteria is that the candidate protein exhibits differential mRNA expression between normal individuals and PsD patients in lesional skin, and also shows difference between lesional and non-lesional sites in PsD patients.

[3]In the 'baseline difference' section, the supporting criterion is that the candidate protein exhibits differing baseline plasma concentrations between individuals who developed PsD during follow-up and those who did not.

[4]In the 'risk stratification' section, the supporting criterion is that the candidate protein has a P-value < 0.05 in the Log-rank test.

[5] In the 'discriminative capacity' section, the supporting criterion is that the candidate protein has an AUC value > 0.5 and a P-value < 0.05 for distinguishing between individuals who developed PsD over twelve years.

*These proteins were not detected in the Olink® Explore 3072 panel and were marked as 'Not available'.

AIF1, allograft inflammatory factor 1; ATP6V1G2, V-type proton ATPase subunit G2; BTN2A1, butyrophilin subfamily 2 member A1; CDSN, corneodesmosin; DDR1, epithelial discoidin domain-containing receptor 1; DXO, decapping and exoribonuclease protein; FAP, prolyl endopeptidase; GCA, grancalcin; HCG22, protein PBMUCL2; HLA-E, HLA class I histocompatibility antigen, alpha chain E; ICAM3, intercellular adhesion molecule 3; IL12B, interleukin-12 subunit beta; IL23R, interleukin-23 receptor; KLK1, kallikrein-1; LTA, lymphotoxin-alpha; LTB, lymphotoxin-beta; MICA/MICB, MHC class I polypeptide-related sequence A/B; PLAU, urokinase-type plasminogen activator; PRSS53, serine protease 53; PRSS8, prostasin; RIGI, antiviral innate immune response receptor RIG-I; STAT3, signal transducer and activator of transcription 3.

Supplementary Table 18. Baseline characteristics after matching

Characteristic	Non-PsD	PsD	*P*-value	Test SMD
N	188	188		
Age (mean (SD))	59.94 (7.42)	59.93 (7.09)	0.989	0.001
Sex = Male (%)	101 (53.7)	101 (53.7)	1.000	<0.001
BMI (mean (SD))	28.50 (4.75)	28.51 (5.12)	0.980	0.003

P-value less than 0.05 suggests a statistically significant difference.
SMD closer to 0 indicates smaller or negligible differences, while larger values suggest more substantial differences between the groups.

Supplementary Table 19. Baseline characteristics adjusted for additional confounding factors

**Characteristic**	**Non-PsD (N=32091)**	**PsD (N = 188)**	***P*-value**
Age (mean (SD))	56.98 (8.19)	59.93 (7.09)	<0.001
Sex = Male (%)	14,457 (45.1)	101 (53.7)	0.021
BMI (mean (SD))	27.46 (4.78)	28.51 (5.12)	0.003
PRS (mean (SD))	-0.17 (1.01)	0.46 (1.17)	<0.001
TDI (mean (SD))	-1.36 (3.08)	-0.70 (3.37)	0.003
General health rating (%)			<0.001
Poor	1,439 (4.5)	18 (9.6)	
Fair	6,528 (20.3)	54 (28.7)	
Good	18,729 (58.4)	99 (52.7)	
Excellent	5,395 (16.8)	17 (9.0)	
Med_BP	7,123 (22.2)	65 (34.6)	<0.001
Med_Chol	5,865 (18.3)	47 (25.0)	0.022
Med_HRT	1,243 (3.9)	5 (2.7)	0.502
Med_Insulin	355 (1.1)	3 (1.6)	0.772
Med_Category (%)			0.006
None	21,342 (67.3)	104 (55.3)	
Single medication	6,411 (20.2)	50 (26.6)	
Two medications	3,670 (11.6)	32 (17.0)	
≥3 medications	277 (0.9)	2 (1.1)	

PRS (Polygenic Risk Scores): A measure of genetic susceptibility to psoriasis, calculated based on multiple genetic variants.
TDI (Townsend Deprivation Index): An index used to assess socio-economic deprivation in a specific geographic area, considering factors such as unemployment, overcrowded housing, and car ownership.
Med_ (Medication Use): Information regarding the medications taken by study subjects, which includes treatments for cholesterol, blood pressure, diabetes, and exogenous hormone therapy.

Supplementary Table 20. Characteristics of PsD patients in exploring the correlation between CDSN and PRSS8 with disease severity

Number	Gender (Male/Female)	Age (Year)	PASI score
1	Male	25	8
2	Male	31	9
3	Male	26	19.5
4	Male	26	22.3
5	Male	30	3.7
6	Male	17	5.4
7	Female	61	6.9
8	Female	54	4.3
9	Male	56	15
10	Male	62	1.5

Supplementary Table 21. Codes used for diagnosis and classification of diseases in the UKB

Diagnosis	ICD10 codes	ICD9 codes	Self-report codes
Field ID	41202 (41204)	41203 (41205)	20002
Psoriasis vulgaris	L40-L400	/	1453
Generalised pustular Psoriasis	L40-L401	/	/
Pustulosis palmaris et plantaris	L40-L403	/	/
Guttate Psoriasis	L40-L404	/	/
Arthropathic Psoriasis	L40-L405	6960	1477
Other Psoriasis	L40-L408	6961	/
Psoriasis, unspecified	L40-L409	/	/
Cutaneous abscess, furuncle and carbuncle	L02	680	/
Cellulitis	L03	681/682	1625
Other local infections of skin and subcutaneous tissue	L08	686	/
Irritant contact dermatitis	L24	692	1669
Dermatitis due to substances taken internally	L27	693	/
Pruritus	L29	698	/
Lichen planus	L43	697	1549
Follicular cysts of skin and subcutaneous tissue	L72	/	/
Seborrhoeic keratosis	L82	/	/
Decubitus ulcer	L89	7070	/
Atrophic disorders of skin	L90	701	/
Other disorders of skin and subcutaneous tissue, not elsewhere classified	L98	709	/

Please refer to the webpage of UK-Biobank for detailed information (https://www.ukbiobank.ac.uk).
UKB, UK Biobank; ICD = International Classification of Diseases.

Supplementary Table 22. Candidate predictors used in clinical machine learning model

Main category	Subcategory	Field ID
Population characteristic (n = 3)	Baseline characteristics (n = 3)	31-0.0, 21022-0.0, 52-0.0
Physical measures (n = 55)	Body composition by impedance (n = 32)	23098-0.0, 23099-0.0, 23100-0.0, 23101-0.0, 23102-0.0, 23104-0.0, 23105-0.0, 23106-0.0, 23107-0.0, 23108-0.0, 23109-0.0, 23110-0.0, 23111-0.0, 23112-0.0, 23113-0.0, 23114-0.0, 23115-0.0, 23116-0.0, 23117-0.0, 23118-0.0, 23119-0.0, 23120-0.0, 23121-0.0, 23122-0.0, 23123-0.0, 23124-0.0, 23125-0.0, 23126-0.0, 23127-0.0, 23128-0.0, 23129-0.0, 23130-0.0
	Body size measures (n = 8)	48-0.0, 49-0.0, 50-0.0, 51-0.0, 3077-0.0, 20015-0.0, 21001-0.0, 21002-0.0
	Blood pressure (n = 3)	102-0.0, 4079-0.0, 4080-0.0
	Bone-densitometry (n = 1)	3082-0.0
	Hand grip strength (n = 2)	46-0.0, 47-0.0
	Spirometry (n = 9)	3059-0.0, 3062-0.0, 3063-0.2, 3064-0.2, 20150-0.0, 20151-0.0, 20256-0.0, 20257-0.0, 20258-0.0
Blood assays (n=59)	Blood biochemistry (n = 28)	30600-0.0, 30610-0.0, 30620-0.0, 30630-0.0, 30640-0.0, 30650-0.0, 30660-0.0, 30670-0.0, 30680-0.0, 30690-0.0, 30700-0.0, 30710-0.0, 30720-0.0, 30730-0.0, 30740-0.0, 30750-0.0, 30760-0.0, 30770-0.0, 30780-0.0, 30790-0.0, 30810-0.0, 30830-0.0, 30840-0.0, 30850-0.0, 30860-0.0, 30870-0.0, 30880-0.0, 30890-0.0
	Blood count (n = 31)	30000-0.0, 30010-0.0, 30020-0.0, 30030-0.0, 30040-0.0, 30050-0.0, 30060-0.0, 30070-0.0, 30080-0.0, 30090-0.0, 30100-0.0, 30110-0.0, 30120-0.0, 30130-0.0, 30140-0.0, 30150-0.0, 30160-0.0, 30170-0.0, 30180-0.0, 30190-0.0, 30200-0.0, 30210-0.0, 30220-0.0, 30230-0.0, 30240-0.0, 30250-0.0, 30260-0.0, 30270-0.0, 30280-0.0, 30290-0.0, 30300-0.0
Urine assays (n = 3)	Urine biochemistry (n = 3)	30510-0.0, 30520-0.0, 30530-0.0
Touchscreen questionnaires (n=169)	Family history (n = 9)	1797-0.0, 1807-0.0, 1835-0.0, 1873-0.0, 1883-0.0, 3526-0.0, 20107-0.0, 20110-0.0, 20111-0.0
	Medication history (n = 55)	2316-0.0, 2335-0.0, 2207-0.0, 2217-0.0, 2227-0.0, 2188-0.0, 2296-0.0, 2306-0.0, 2247-0.0, 2257-0.0, 3393-0.0, 2443-0.0, 2453-0.0, 2463-0.0, 2473-0.0, 6150-0.0, 6152-0.0, 2492-0.0, 6154-0.0, 6155-0.0, 6179-0.0, 6149-0.0, 6159-0.0, 1920-0.0, 1930-0.0, 1940-0.0, 1950-0.0, 1960-0.0, 1970-0.0, 1980-0.0, 1990-0.0, 2000-0.0, 2010-0.0, 2020-0.0, 2030-0.0, 2040-0.0, 2050-0.0, 2060-0.0, 2070-0.0, 2080-0.0,

	2090-0.0, 2100-0.0, 6145-0.0, 20127-0.0, 87-0.0, 134-0.0, 135-0.0, 20008-0.0, 20009-0.0, 137-0.0, 92-0.0, 136-0.0, 3079-0.0, 20010-0.0, 20011-0.0
Sociodemographics (n = 3)	6138-0.0, 6142-0.0, 21000-0.0
Lifestyle and environment (n = 94)	1558-0.0, 1568-0.0, 1578-0.0, 1588-0.0, 1598-0.0, 1608-0.0, 1618-0.0, 1628-0.0, 20117-0.0, 1289-0.0, 1299-0.0, 1309-0.0, 1319-0.0, 1329-0.0, 1339-0.0, 1349-0.0, 1359-0.0, 1369-0.0, 1379-0.0, 1389-0.0, 1408-0.0, 1418-0.0, 1428-0.0, 1438-0.0, 1448-0.0, 1458-0.0, 1468-0.0, 1478-0.0, 1488-0.0, 1498-0.0, 1508-0.0, 1518-0.0, 1528-0.0, 1538-0.0, 1548-0.0, 6144-0.0, 1110-0.0, 1120-0.0, 1130-0.0, 1140-0.0, 1150-0.0, 2237-0.0, 864-0.0, 874-0.0, 884-0.0, 894-0.0, 904-0.0, 924-0.0, 943-0.0, 971-0.0, 981-0.0, 1070-0.0, 1080-0.0, 1090-0.0, 1100-0.0, 6162-0.0, 6164-0.0, 22033-0.0, 22034-0.0, 22035-0.0, 22036-0.0, 22037-0.0, 22038-0.0, 22039-0.0, 22040-0.0, 2139-0.0, 2149-0.0, 2159-0.0, 1160-0.0, 1170-0.0, 1180-0.0, 1190-0.0, 1200-0.0, 1210-0.0, 1220-0.0, 1239-0.0, 1249-0.0, 1259-0.0, 1269-0.0, 1279-0.0, 20116-0.0, 20160-0.0, 1050-0.0, 1060-0.0, 1717-0.0, 1727-0.0, 1737-0.0, 1747-0.0, 1757-0.0, 2267-0.0, 2277-0.0, 1031-0.0, 2110-0.0, 6160-0.0
Early life factors (n = 8)	1647-0.0, 1677-0.0, 1687-0.0, 1697-0.0, 1707-0.0, 1767-0.0, 1777-0.0, 1787-0.0

For the rest predictors please refer to the webpage of UK-Biobank for detailed information (https://www.ukbiobank.ac.uk).

Supplementary Table 23. Hyperparameter space explored for LightGBM classifier

Hyperparameters	Search space	Step	Final choice
n_estimators	100 to 1000	100	500
max_depth	3 to 30	3	15
num_leaves	10 to 100	10	10
colsample_bytree	0.7 to 1	0.05	0.7
subsample	0.7 to 1	0.05	0.7
learning_rate	1e-5 to 1e-1	*10	1e-2

Detailed explanation for each parameters please refer to: https://lightgbm.readthedocs.io/en/v3.3.2/Parameters.html.

Supplementary Table 24. The information of the reagents used in this study

**Reagent**	**Source**	**Identifier**
In vivo si-PRSS8	RIBOBIO	N/A
In vivo si-CDSN	RIBOBIO	N/A
In vivo si-NC	RIBOBIO	N/A
CDSN antibody	Proteintech	Cat No. 13184-1-AP
PRSS8 antibody	Proteintech	Cat No. 15527-1-AP
IL-17A antibody	Proteintech	Cat No. 66148-1-Ig
RORγt antibody	Bioss	Cat No. Bs-10647R
β-actin antibody	Proteintech	Cat No. 66009-1-Ig
K14 antibody	Proteintech	Cat No. 60320-1-Ig
CD45 PE/Cyanine5 antibody	Biolegend	Cat No. 103109
CD3 APC/Cyanine7 antibody	Biolegend	Cat No. 100221
CD4 FITC antibody	Biolegend	Cat No. 100405
IL-17A PE antibody	Biolegend	Cat No. 506903
Fixable Viability Dye	eBioscience	Cat No. 65-0863-18
Multi-rAb HRP-Goat Anti-Mouse Recombinant Secondary Antibody (H+L)	Proteintech	Cat No. RGAM001
Multi-rAb HRP-Goat Anti-Rabbit Recombinant Secondary Antibody (H+L)	Proteintech	Cat No. RGAR001
Multi-rAb CoraLite® Plus 488-Goat Anti-Rabbit Recombinant Secondary Antibody (H+L)	Proteintech	Cat No. RGAR002
Multi-rAb CoraLite® Plus 594-Goat Anti-Mouse Recombinant Secondary Antibody (H+L)	Proteintech	Cat No. RGAM004
Cell stimulation cocktail (plus protein transport inhibitors)	Thermofisher	Cat No. 00-4975-93
Imiquimod cream	Shichuan MedShine Pharmaceuticals	N/A

Supplementary Table 25. STROBE-MR checklist of recommended items to address in reports of Mendelian randomization studies

Item No.	Section	Checklist item	Page No.
1	**TITLE and ABSTRACT**	Indicate Mendelian randomization (MR) as the study's design in the title and/or the abstract if that is a main purpose of the study	1
	**INTRODUCTION**		
2	**Background**	Explain the scientific background and rationale for the reported study. What is the exposure? Is a potential causal relationship between exposure and outcome plausible? Justify why MR is a helpful method to address the study question	4
3	**Objectives**	State specific objectives clearly, including pre-specified causal hypotheses (if any). State that MR is a method that, under specific assumptions, intends to estimate causal effects	5
	**METHODS**		
4	**Study design and data sources**	Present key elements of the study design early in the article. Consider including a table listing sources of data for all phases of the study. For each data source contributing to the analysis, describe the following:	
		a) Setting: Describe the study design and the underlying population, if possible. Describe the setting, locations, and relevant dates, including periods of recruitment, exposure, follow-up, and data collection, when available.	24
		b) Participants: Give the eligibility criteria, and the sources and methods of selection of participants. Report the sample size, and whether any power or sample size calculations were carried out prior to the main analysis	24
		c) Describe measurement, quality control and selection of genetic variants	25
		d) For each exposure, outcome, and other relevant variables, describe methods of assessment and diagnostic criteria for diseases	24
		e) Provide details of ethics committee approval and participant informed consent, if relevant	25
5	**Assumptions**	Explicitly state the three core IV assumptions for the main analysis (relevance, independence and exclusion restriction) as well assumptions for any additional or sensitivity analysis	25-26
6	**Statistical methods: main analysis**	Describe statistical methods and statistics used	
		a) Describe how quantitative variables were handled in the analyses (i.e., scale, units, model)	26-27
		b) Describe how genetic variants were handled in the analyses and, if applicable, how their weights were selected	25
		c) Describe the MR estimator (e.g. two-stage least squares, Wald ratio) and related statistics. Detail the included covariates and, in case of two-sample MR, whether the same covariate set was used	26

			for adjustment in the two samples	
		d)	Explain how missing data were addressed	27
		e)	If applicable, indicate how multiple testing was addressed	
7	**Assessment of assumptions**		Describe any methods or prior knowledge used to assess the assumptions or justify their validity	25
8	**Sensitivity analyses and additional analyses**		Describe any sensitivity analyses or additional analyses performed (e.g. comparison of effect estimates from different approaches, independent replication, bias analytic techniques, validation of instruments, simulations)	27-28
9	**Software and pre-registration**			
		a)	Name statistical software and package(s), including version and settings used	29
	**RESULTS**			
10	**Descriptive data**			
		a)	Report the numbers of individuals at each stage of included studies and reasons for exclusion.	24
		b)	Report summary statistics for phenotypic exposure(s), outcome(s), and other relevant variables (e.g. means, SDs, proportions)	6
		c)	If the data sources include meta-analyses of previous studies, provide the assessments of heterogeneity across these studies	6
		d)	For two-sample MR:<br>i. Provide justification of the similarity of the genetic variant-exposure associations between the exposure and outcome samples<br>ii. Provide information on the number of individuals who overlap between the exposure and outcome studies	6
11	**Main results**			
		a)	Report the associations between genetic variant and exposure, and between genetic variant and outcome, preferably on an interpretable scale	6
		b)	Report MR estimates of the relationship between exposure and outcome, and the measures of uncertainty from the MR analysis, on an interpretable scale	6
		c)	If relevant, consider translating estimates of relative risk into absolute risk for a meaningful time period	
		d)	Consider plots to visualize results (e.g. forest plot, scatterplot of associations between genetic variants and outcome versus between genetic variants and exposure)	7
12	**Assessment of**			

	**assumptions**			
		a)	Report the assessment of the validity of the assumptions	6
		b)	Report any additional statistics (e.g., assessments of heterogeneity across genetic variants, such as I^2, Q statistic or E-value)	6
13	**Sensitivity analyses and additional analyses**			
		a)	Report any sensitivity analyses to assess the robustness of the main results to violations of the assumptions	6-7
		b)	Report results from other sensitivity analyses or additional analyses	6-7
		c)	Report any assessment of direction of causal relationship (e.g., bidirectional MR)	6
		d)	When relevant, report and compare with estimates from non-MR analyses	8-10
	**DISCUSSION**			
14	**Key results**		Summarize key results with reference to study objectives	15
15	**Limitations**		Discuss limitations of the study, taking into account the validity of the IV assumptions, other sources of potential bias, and imprecision. Discuss both direction and magnitude of any potential bias and any efforts to address them	18
16	**Interpretation**			
		a)	Meaning: Give a cautious overall interpretation of results in the context of their limitations and in comparison with other studies	20
		b)	Mechanism: Discuss underlying biological mechanisms that could drive a potential causal relationship between the investigated exposure and the outcome, and whether the gene-environment equivalence assumption is reasonable. Use causal language carefully, clarifying that IV estimates may provide causal effects only under certain assumptions	20
		c)	Clinical relevance: Discuss whether the results have clinical or public policy relevance, and to what extent they inform effect sizes of possible interventions	21
17	**Generalizability**		Discuss the generalizability of the study results (a) to other populations, (b) across other exposure periods/timings, and (c) across other levels of exposure	22
	**OTHER INFORMATION**			
18	**Funding**		Describe sources of funding and the role of funders in the present study and, if applicable, sources of funding for the databases and original study or studies on which the present study is based	2
19	**Data and data**		Provide the data used to perform all analyses or report where and how the data can be accessed,	37

	**sharing**	and reference these sources in the article. Provide the statistical code needed to reproduce the results in the article, or report whether the code is publicly accessible and if so, where	
20	**Conflicts of Interest**	All authors should declare all potential conflicts of interest	37

This checklist is copyrighted by the Equator Network under the Creative Commons Attribution 3.0 Unported (CC BY 3.0) license.

1. Skrivankova VW, Richmond RC, Woolf BAR, Yarmolinsky J, Davies NM, Swanson SA, et al. Strengthening the Reporting of Observational Studies in Epidemiology using Mendelian Randomization (STROBE-MR) Statement. JAMA. 2021.
2. Skrivankova VW, Richmond RC, Woolf BAR, Davies NM, Swanson SA, VanderWeele TJ, et al. Strengthening the Reporting of Observational Studies in Epidemiology using Mendelian Randomisation (STROBE-MR): Explanation and Elaboration. BMJ. 2021;375:n2233.
